# A three-photon head-mounted microscope for imaging all layers of visual cortex in freely moving mice

**DOI:** 10.1038/s41592-022-01688-9

**Published:** 2022-11-28

**Authors:** Alexandr Klioutchnikov, Damian J. Wallace, Juergen Sawinski, Kay-Michael Voit, Yvonne Groemping, Jason N. D. Kerr

**Affiliations:** Department of Behavior and Brain Organization, Max Planck Institute for Neurobiology of Behavior—caesar, Bonn, Germany

**Keywords:** Neuroscience, Imaging, Mouse, Multiphoton microscopy

## Abstract

Advances in head-mounted microscopes have enabled imaging of neuronal activity using genetic tools in freely moving mice but these microscopes are restricted to recording in minimally lit arenas and imaging upper cortical layers. Here we built a 2-g, three-photon excitation-based microscope, containing a *z*-drive that enabled access to all cortical layers while mice freely behaved in a fully lit environment. The microscope had on-board photon detectors, robust to environmental light, and the arena lighting was timed to the end of each line-scan, enabling functional imaging of activity from cortical layer 4 and layer 6 neurons expressing jGCaMP7f in mice roaming a fully lit or dark arena. By comparing the neuronal activity measured from populations in these layers we show that activity in cortical layer 4 and layer 6 is differentially modulated by lit and dark conditions during free exploration.

## Abstract (web summary)

A lightweight three-photon miniature microscope allows imaging neuronal activity throughout the cortex in freely moving mice.

## Main

Recently developed multiphoton head-mounted microscopes^1–4^ can utilize, with single-cell resolution, the vast range of genetically encoded tools^5^ used for recording neuronal activity in freely behaving mice (for reviews, see refs. ^6–8^). Two-photon excitation-based head-mounted microscopes are restricted to imaging neuronal activity in upper cortical layers^1,2,4^, whereas three-photon excitation (3PE)-based head-mounted microscopes, while removing this imaging-depth limitation^9^, have so far been too physically restrictive to take full advantage of mouse-based molecular tools. To gain access to larger numbers of neurons and remove the need to interfere with animal behavior when changing the focal plane, remote focusing has been applied to several two-photon excitation-based head-mounted microscopes^2,4^. While this has increased focusing range by several hundred microns and allowed volume imaging at low frame rates, acquisition is still limited to the upper cortical layers and the microscope weight has markedly increased^2^. In addition, as mice actively sense their environment using vision^10,11^, utilizing their full behavioral repertoire requires a fully lit visual environment, which is problematic for head-mounted microscopes using photon multiplier tubes owing to their sensitivity^1,2,4,9,12^. Ideally, a head-mounted multiphoton microscope could image from all cortical layers, could shift imaging depth at will between layers and be usable in a fully lit environment to allow the animals to access their full sensory repertoire^10,11^. Here we designed a head-mounted three-photon excitation microscope^9^ capable of imaging activity from all cortical layers in mice, with single-cell resolution, which contained a modified detector system that allowed imaging in a lit environment.

## Results

### Lightweight three-photon microscope with remote focusing

**Fig. 1:**
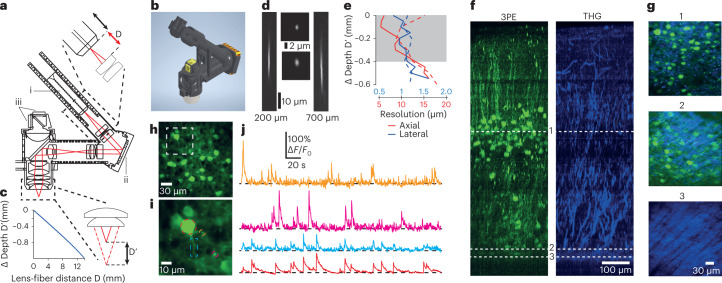
A lightweight miniature three-photon fiberscope with *z*-drive for imaging in freely moving mice. **a**, Microscope schematic showing the ferrule used to hold the fiber tip (i), the MEMS scanner (ii) and the on-board detectors (iii). Shifting the fiber tip through distance D (top insert) shifts the imaging plane by distance D′ (bottom insert). Microscope optical parts are shown in Supplementary Fig. 1. **b**, Three-dimensional miniature microscope model. **c**, Zemax-simulation of relationship between the lens–fiber tip distance (distance D in **a**) and corresponding imaging plane depth (distance D′ in **a**). **d**, Side projections (left and right) and single images (center), acquired with the *z*-drive set to the indicated focus depths, of a 500-nm fluorescent bead imaged with the extended z-range version of the miniature microscope. The 10-µm scale applies to both side projections and the 2 µm scale to both single images. **e**, Measured axial (red) and lateral (blue) resolution of both the high-resolution (solid) and extended *z*-range (dashed) versions of the miniature microscope as a function of changing depth of the imaging plane by fiber movement. Measurements made using 0.5 µm fluorescent beads. **f**, Side projections showing jGCaMP7f-labeled neurons (left) and third-harmonic generation signal (right) acquired from an anesthetized mouse with the microscope mounted on an external micromanipulator. Dashed lines indicate the imaging planes for the corresponding images shown in **g**. Scale in the right image applies to both images. **g**, Individual images from the data in **f**. Scale in image 3 applies to all images. **h**, Image of a population of layer 4 neurons in an anesthetized mouse imaged with the high-resolution version of the microscope. Region in dashed box shown in **i**. Laser power 8.8 mW, imaging depth 376 µm. **i**, Enlarged section of the image in **h**, showing the neuronal soma (red dashed) and dendrites (blue, orange and magenta dashed) from which the calcium kinetic traces in **j** were recorded. Note that the dendrite in the blue dashed box is a basal dendrite of the neuronal soma in the red dashed circle. **j**, Calcium kinetic traces from the labeled structures in **i**. Scale bar applies to all traces.

The design specifications for the microscope were firstly, that it had focusing capability with a sufficient range to cover the entire cortical depth of the mouse; secondly, that the optical pathway maintained single-cell resolution across the entire focus range; and thirdly, that it operated remotely to allow changes in the imaging plane in a freely behaving animal without the need to disturb animal behavior. To allow focusing, we designed an optical system which, like previous bench-top designs^13^, changed the beam collimation properties (defocus) to produce changes in the working distance (Fig. 1a and Supplementary Fig. 1a). As there is a tradeoff between focus range and axial resolution, we made the microscope design modular (Fig. 1b and Supplementary Fig. 1b) to allow the optics to be configured to suit different experimental requirements, resulting in both extended *z*-range and higher-resolution configurations.

In the extended *z*-range configuration, the optical system had a simulated working distance ranging from 1.1 to 1.9 mm below the objective, with the simulation predicting that imaging was diffraction-limited over (>700 µm of the total range Supplementary Fig. 1c) with an excitation numerical aperture (NA = 0.4) suitable for cellular resolution^14^. To generate the necessary defocus required for remotely changing the working distance^15^, we developed a lightweight mechanical system that exploited the divergence of the beam when exiting the fiber tip, enabling changes in the working distance as the distance between the fiber tip and the collimation lens changed (Fig. 1a and Supplementary Fig. 1d). A 12-mm movement combined with a collimation lens with a 6-mm effective focal length (EFL) produced zemax-simulated *z*-range >700 µm (Fig. 1c). With this optical configuration, the field of view (FOV) was >300 µm (limited by the scanner specifications), with the measured resolution over the full range of focus ranging between 1.0–1.2 µm lateral and 9–19 µm axial (full width half maximum (FWHM); Fig. 1d,e). This approach added minimal additional weight (<100 mg), and the smaller specified EFL of the objective lens (1.8 mm versus 3 mm in a previous design^9^) reduced the weight of the optical system compared to our previous miniature 3PE microscope (720 mg versus >2 g in the previous design^9^). Using this design, the fully assembled microscope weighed ~2 g. The 3PE allowed imaging of neuronal somata and dendrites labeled with the genetically encoded calcium indicator jGCaMP7f as well as neuronal and vascular structures with third-harmonic generation (THG)^16,17^ from cortical layer 1 to the corpus callosum in the mouse (Fig. 1f,g and Supplementary Fig. 2), and the entire cortical mantel was accessible using the remote *z*-drive mechanism (Supplementary Movie 1). The high-resolution configuration uses alternative lenses in three positions in the microscope optical path (Supplementary Fig. 3), all of which, for both extended *z*-range and high-resolution configuration, are commercially available. In the high-resolution configuration the microscope had a measured axial resolution of ~6 µm over a *z*-range of around 150 µm (Fig. 1e). This configuration had a FOV of ~200 µm, and allowed functional imaging from basal and apical dendrites as well as neuronal somata (Fig. 1h–j). We also tested an electrically tunable lens^18^ (ETL; Supplementary Fig. 4), allowing the imaging plane to be moved over a range of ~400 µm, with measured axial resolution <17 µm over the full range (FWHM; Supplementary Fig. 4d). While the ETL provided fast access to any imaging plane within the range, and potential for multiplane imaging^4,19^, it could not utilize the full potential range of working distance movement provided by the designed optical pathway and also increased the total microscope weight by ~40% (>800 mg). In summary, the microscope design allows imaging of neuronal somata and dendrites throughout the thickness of the mouse cortex, with remote focusing to allow adjustment of the imaging plane depth in stationary (Supplementary Video 1) or freely moving mice (Supplementary Video 2) without the need to interfere with animal behavior.

### Imaging activity in layer 4 and 6 in freely moving mice

**Fig. 2:**
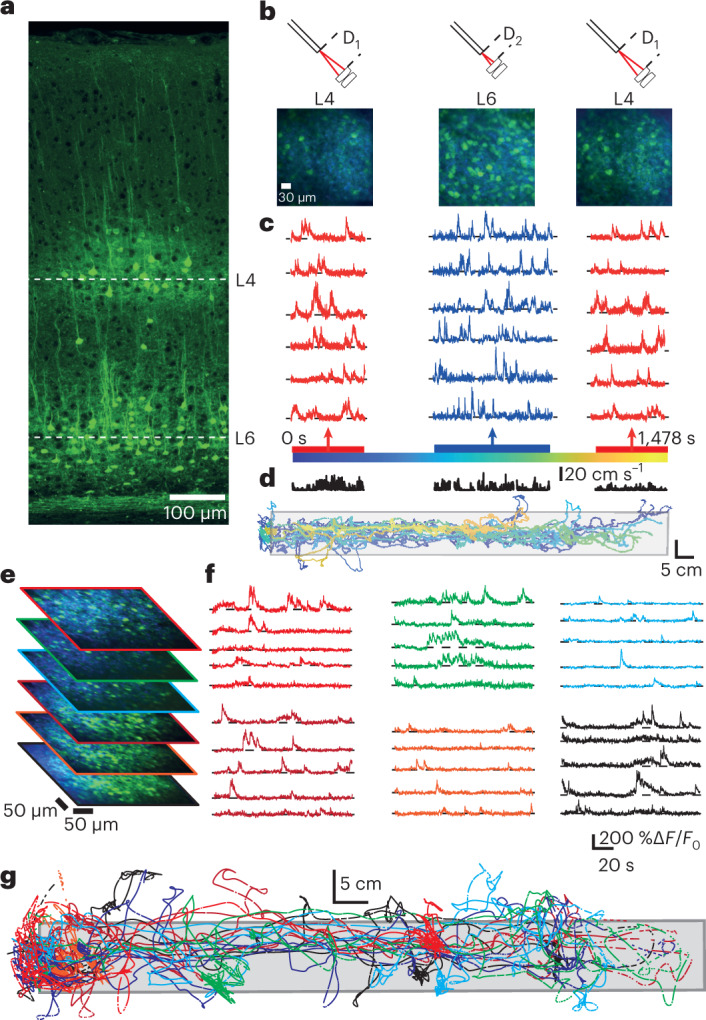
Imaging different neuronal populations in a freely moving mouse using the remote focus mechanism. **a**, Histological section showing jGCaMP7f labeling in layers 4 and 6 of mouse primary visual cortex. **b**, Overview images (lower) and schematics (upper) of the fiber tip to lens distances (distance D in Fig. 1a) for imaging planes sequentially acquired in L4 and L6 for which example neuronal Ca^2+^-transients are shown in **c**. **c**, Example Ca^2+^-fluorescence traces from sequential imaging of neuronal populations in layers 4 (red) and 6 (blue) in the same behavioral session from one animal. Corresponding overview images shown in **b**. Color coding in the time scale (bottom) corresponds with the color-coded position of the animal on the linear track shown in **d**. Laser power for the first and second Ca^2+^-imaging movies in layer 4 were 27 and 30 mW, respectively, and 44 mW for the movie in layer 6. **d**, Animal position on the linear track during the behavioral session in which the data in **c** were acquired. Color coding corresponds with the color-coded time scale in **c**. **e**, Overview images and example Ca^2+^-fluorescence traces from sequential imaging of multiple planes of labeled neurons in layer 4 acquired in one behavioral session. Colored border on overview images corresponds to the color of the example Ca^2+^-traces. Scale applies to all Ca^2+^-traces. Laser power for the six Ca^2+^-imaging movies shown were respectively (top (red) to bottom (black)) 26.5, 31, 33, 37, 38 and 56 mW. **g**, Position of the animal on the track during the acquisitions shown in **e** and **f**. Color coding as in **e**,**f**.

To confirm the range and stability of the microscope and mechanical *z*-drive mechanism, we created a mouse line expressing cre recombinase both in layer 4 (L4)^20^ and 6 (L6)^21^. Using an adeno-associated virus encoding cre-dependent jGCaMP7f we could drive indicator expression selectively in those cortical layers (Fig. 2a). Using the extended *z*-range configuration of the microscope, we could sequentially measure calcium transients from neuronal populations in L4 and L6 in single imaging sessions (Fig. 2b–d; *N* = 9 L4 populations from 3 animals and *N* = 9 L6 populations from 4 animals), or measure from multiple imaging planes within a layer (Fig. 2e–g and Supplementary Fig. 5), and could make repeated measurements from these cortical layers in sessions spread over multiple days (Supplementary Fig. 6; *N* = 9 sessions total, from 4 animals, number of imaging days 2.3 ± 1.3 (mean ± s.d.), range 1 to 4, average days post window implant 5.7 ± 2.7 days (mean ± s.d.), range 2 to 10). In these experiments there was an average of 34.2 ± 15.7 neurons per FOV for imaging in L4 (mean ± s.d., range 20 to 60, *N* = 9 FOVs) and 98.7 ± 25.8 in L6 (mean ± s.d., range 68 to 141, *N* = 9 FOVs). Average laser power post-objective used to image populations in layer 4 was 19.6 ± 6.3 mW (mean ± s.d., range 11.5–30 mW, 14 Ca^2+^-imaging movies from 3 animals) and in layer 6 was 41.6 ± 7.4 mW (mean ± s.d., range 27–53 mW, 17 Ca^2+^-imaging movies from 3 animals). During imaging sessions, animals were freely exploring a linear track (96.0 × 9.7 cm, length × width), being active on average for 26.8 ± 13.5% of the time (mean ± s.d., *N* = 4 animals). Average movement path length per Ca^2+^-imaging movie was 661.9 ± 299.3 cm (mean ± s.d., range 142.3 to 1,582.7 cm, movie duration 282 s, *N* = 30 Ca^2+^-imaging movies from 18 populations) with an average velocity (while moving) of 9.0 ± 0.85 cm s^−1^ (mean ± s.d., range 7.8 to 10.5 cm s^−1^, *N* = 30 Ca^2+^-imaging movies from 18 populations). During free exploration (Supplementary Videos 3 and 4) frame-wise lateral pixel shifts were typically <5 pixels (Supplementary Fig. 7 and Supplementary Text), similar to previous head-mounted microscopes^1,2,4,9,12^. Excluded data (typical reasons: excessive image motion, entanglement of the optical fiber and electronic cables, and image brightness fluctuation owing to shifting of the immersion solution) was <3% of the recorded data for the majority of Ca^2+^-imaging movies (20 out of 31 movies, average data rejection for all movies 10.3 ± 17.6% (mean ± s.d.), range 0 to 72.1%, *N* = 31 Ca^2+^-imaging movies). We quantified the change in resting fluorescence of the imaged neurons and neuropil occurring over each acquired Ca^2+^-imaging movie as a measure of laser-induced tissue damage (Supplementary Fig. 8). Average neuropil baseline traces from L4 and L6 (Supplementary Fig. 8c) had median decay coefficients of 1.38 × 10^−4^ ± 4.20 × 10^−3^ and 5.18 × 10^−4^ ± 8.06 × 10^−4^ respectively when fitted with an exponential decay (median ± s.d., *N* = 15 L4 and 20 L6 Ca^2+^-imaging movies), and were not significantly different from zero (*P* = 0.38 and 0.18 for L4 and L6, right-tailed rank sum test). This showed that the neuropil was neither systematically increasing nor decreasing its fluorescence over the movies, consistent with minimal or no photobleaching or photodamage. The same was true for exponential fits to neuronal fluorescence (Supplementary Fig. 8c; median ± s.d. decay coefficients for L4 and L6 respectively, 5.92 ×10^−5^ ± 1.29 ×10^−3^ and 4.17 ×10^−4^ ± 1.49 ×10^−3^, *N* = 469 and 1,295 neurons, *P* = 0.46 and 0.25, right-tailed rank sum test). As excessive irradiation during multiphoton imaging has been shown to be associated with a progressive increase in soma fluorescence^22,23^, we also quantified mean baseline soma brightness (20th percentile fluorescence) during the first and last 10% of the Ca^2+^-imaging movies (Supplementary Fig. 8d). We did not observe any systematic increase in soma brightness for neurons in either L4 or L6 (*N* = 469 and 1,265 neurons, *P* = 0.9 and 1.0 for L4 and L6 respectively, one-sided Wilcoxon signed rank test), indicating that the imaging was not causing photodamage. Also consistent with this, we routinely recorded neuronal activity from both L4 and L6 neuronal populations over multiple days (Supplementary Fig. 6).

### Imaging neuronal activity in a lit arena

**Fig. 3:**
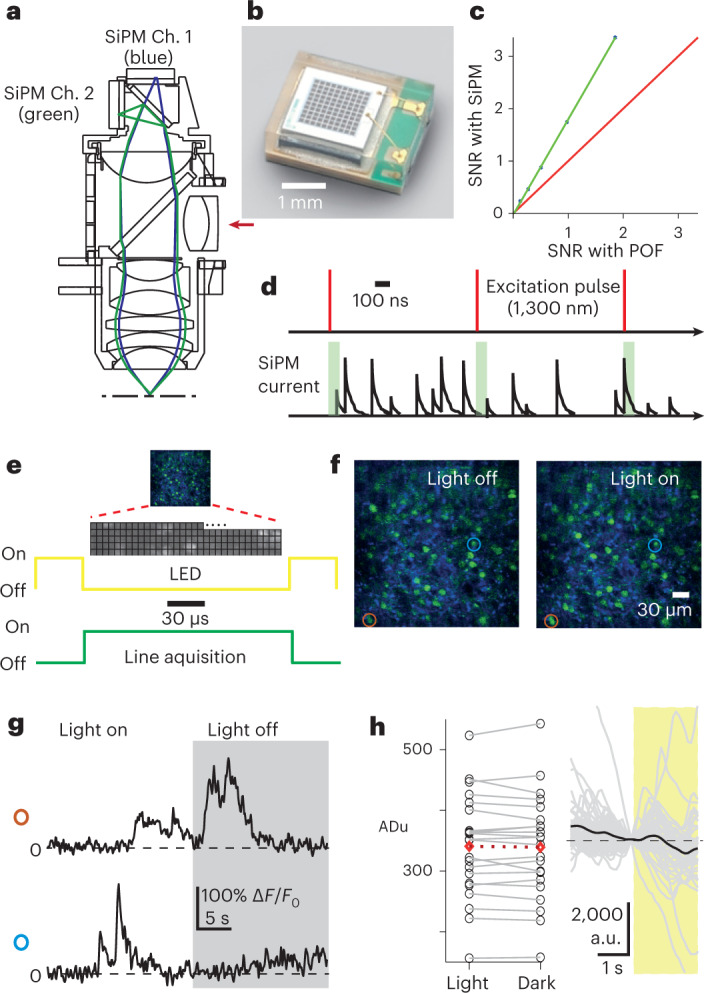
Adaptions allowing imaging in a lit environment. **a**, Microscope schematic highlighting the detector system, showing light paths and on-board detectors for green (green) and third-harmonic generation signal (blue) channels (Ch.). Scanner and *z*-drive optics omitted. The direction of the excitation path is indicated by the red arrow. **b**, Photograph of one of the on-board detectors (SiPM). **c**, Quantification of image signal-to-noise ratio (SNR) using SiPM and remote PMTs coupled via a POF. **d**, Schematic of pixel-wise timing of excitation pulses (top) and emitted fluorescence integration window (bottom, green boxes) used for dark count rejection. **e**, Schematic illustration of the line-wise image acquisition and timing of the environmental lighting activation (LED, yellow) and acquisition of a line in the image (line acquisition, green). **f**, Example images (average of 50 frames) acquired with environmental lighting off (left) or on (right). Data for **f** and **g** from neurons in layer 6, laser power 27 mW. **g**, Example Ca^2+^-fluorescence traces from the neurons indicated in **f** during a transition from the environmental lighting being on to off. **h**, Average pixel intensity over the whole frame from green channel data one frame before (Light) and one frame after (Dark) transitions from light to dark (left) and fluorescence traces from each neuron (gray) from one example recording during a transition from dark to light. Mean of all neuronal traces shown in black. Traces have been normalized to the time point one frame before the time the environmental lights were activated and have been denoised. Open circles in the left panel show data from individual transitions, red diamond represents the mean (*N* = 22 transitions, *P* = 1, two-sample Kolmogorov–Smirnov test). Data in left panel presented as raw analog-to-digital units (ADu), data in the right panel as arbitrary units (a.u.) after denoising.

**Fig. 4:**
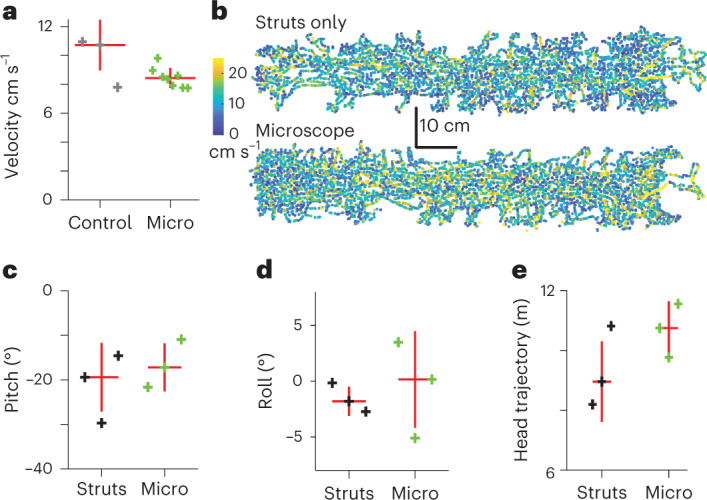
Behavioral characteristics when carrying the miniature microscope. **a**, Average animal velocity with (green crosses) and without microscope (gray crosses). Data with microscope is from all animals imaged (*N* = 8 animals) and data without microscope from a separate set of naive animals (*N* = 3 animals). **b**, Example animal trajectories during one 20-min behavioral session for an animal carrying only position-tracking struts (top) or the full miniature microscope (bottom), with position on the track color-coded by velocity. Both traces are from the same animal, but acquired 3 days apart. Velocity color bar and scale apply to both trajectories. Data representative of three datasets using tracking struts only and 11 datasets with the miniature microscope. **c**, Median head pitch for the same three animals carrying either the tracking struts only (black) or the full miniature microscope (green). **d**, Median head roll, data as for **c**. **e**, Head trajectory data as in **c**,**d**.

To reduce the constraints on animal behavior^3^ of previous miniature microscope designs^1,4,9,24^ and simultaneously to increase efficiency of light collection, we replaced the stiff plastic optical fiber (POF, NA = 0.63, 1 mm diameter) with a two-channel detector system mounted on the microscope (Fig. 3a and Supplementary Fig. 9). These compact and lightweight (2 × 3 mm^2^, 5 mg) silicon photomultipliers (SiPMs; Fig. 3b) have a large detection area (1.3 × 1.3 mm^2^) and high detection efficiency (>60%) and gain (>10^6^). Under matched imaging conditions, the SiPMs achieved a higher signal-to-noise ratio than the POF detection system (factor 1.8 higher; Fig. 3c) over a larger FOV (Supplementary Fig. 9). Cabling for the SiPMs consisted of two thin co-axial cables (<400 μm diameter), which required less force to bend than the POF (bending force for the SiPM co-axial cables or the POF through for a 90° arc with a bend diameter of 10 cm, 1.6 and 160 mN, respectively), making it easier for the animal to move. Unlike gallium arsenide photomultiplier tubes (PMTs)^9,12,25^ SiPMs were resilient to exposure to stray light. To collect mainly emitted fluorescence generated by the excitation pulses and to reduce the high level of dark counts (~10^5^ counts × mm^−1^s^−1^) generated by these SiPMs we integrated the fluorescence signal in a short time window after each laser pulse^9^ (Fig. 3d; maximum 1 MHz excitation pulse rate, 50 ns integration window, rejection ratio 20 times for 1 MHz pulse rate). We also timed the LED lighting of the experimental arena to switch on only during the interval between acquisition of each line (3 kHz) that forms the full frame (Fig. 3e), a flicker rate well above what can be detected by rodents^26,27^. Together this allowed imaging of the same neuronal populations during either fully lit (35 lm m^−2^) or darkened (~0 lm m^−2^) conditions (Fig. 3f–h) without significantly changing the detected fluorescence in the image (Fig. 3h; average pixel intensity over the full frame one frame before and after light–dark transition, *N* = 22 transitions from 3 animals, *P* = 1, two-sample Kolmogorov–Smirnov test). Further, Ca^2+^-fluorescence traces recorded from labeled structures imaged across a transition from light to darkness or darkness to light showed no artifacts in the fluorescence traces (Fig. 3h, Supplementary Fig. 10 and Supplementary Video 5), apart from an occasional transient re-synchronization artifact from environmental lighting activation (mean ± s.d. number of frames with artifacts 1.9 ± 4.8, range 0 to 21, *N* = 20 dark to light transitions from 3 animals). To quantify the effect of carrying the microscope on animal behavior and mobility we compared velocities of animals exploring the track between naive mice carrying nothing and the mice carrying the microscope. Median velocity with and without the microscope was not significantly different (Fig. 4a, b, median ± s.d., naive mice, 10.72 ± 1.75 cm s^−1^, *N* = 3 animals, carrying microscope 8.43 ± 0.70 cm s^−1^, *N* = 8 animals, *P* = 0.28, Wilcoxon rank sum test) and velocity distributions were similar (Supplementary Fig. 11a and Supplementary Text). To quantify the effect of the microscope on head orientation, we made recordings from animals carrying the microscope or carrying only the position-tracking struts. Median values for head pitch (Fig. 4c and Supplementary Fig. 11b), head roll (Fig. 4d and Supplementary Fig. 11c) and head trajectory (Fig. 4b,e) were all not significantly different between the two groups (median ± s.d., pitch, microscope −17.20 ± 5.03°, struts −19.41 ± 8.06°, *P* = 0.7; roll, microscope 0.17 ± 4.30°, struts −1.50 ± 1.29 °, *P* = 0.7; head trajectory, microscope 10.74 ± 0.89 m, struts 8.95 ± 1.35 m, *P* = 0.4; *N* = 3 animals in all cases, Wilcoxon rank sum test in all cases). Together, these experiments show that the behavior of the mice was not significantly perturbed while carrying the miniature three-photon microscope.

### L4 and L6 are differentially modulated in light and dark

**Fig. 5:**
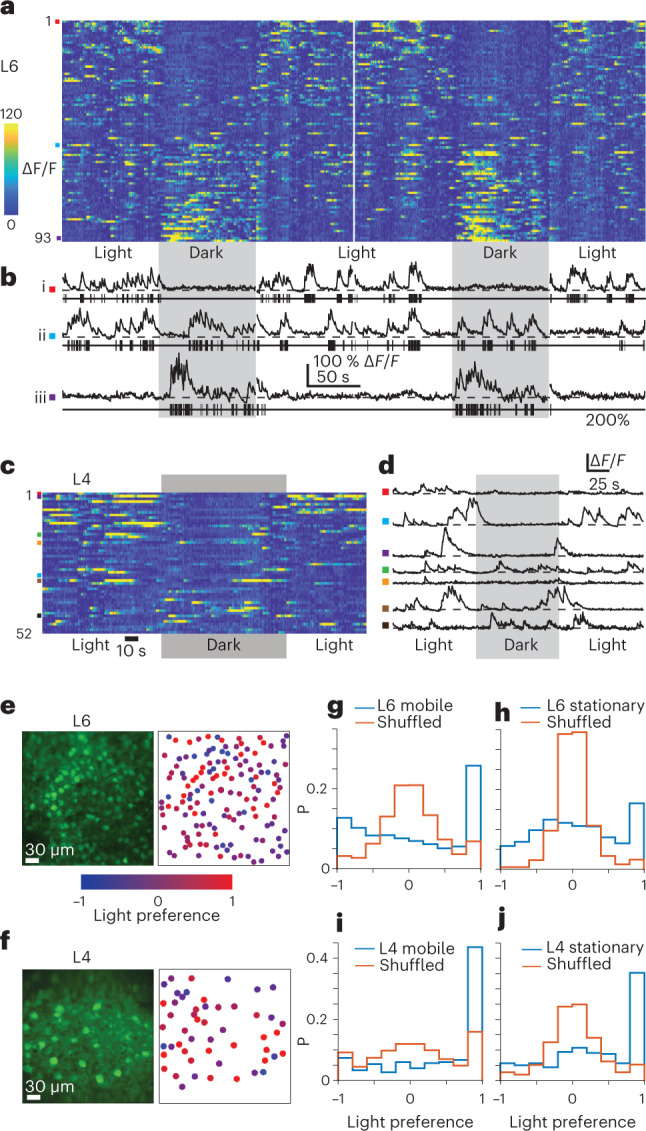
Differential modulation of neurons in L4 and L6 by light and dark. **a**, Ca^2+^-fluorescence traces for all neurons in an example L6 population in two consecutively acquired Ca^2+^-imaging movies. Both Ca^2+^-imaging movies have transitions between environmental lighting being on (Light) and off (Dark, gray boxes). Color-coded fluorescence traces are sorted by neuronal light–dark preference index. Laser power 38 mW. **b**, Example fluorescence traces from three neurons in **a**. Black ticks below example Ca^2+^-traces indicate inferred action potential firing. Time scale in the individual Ca^2+^-traces applies to both the color-coded plot and the individual traces. Fluorescence scale bar applies to all individual Ca^2+^-traces. **c**, Color-coded fluorescence traces as in **a** but for an example population in layer 4 from the same imaging session in the same animal as the data in **a** and **b**. **d**, Example Ca^2+^-fluorescence traces from a L4 population with periods where the environmental lighting is on or off (gray box). Laser power 18 mW. **e**, Overview image of an example neuronal population in layer 6 (left) and the same population of neurons color-coded by light–dark preference (right). **f**, As in **e**, but for an example population in layer 4. **g**, Distributions of light–dark preference index (blue) for all recorded neurons in L6 in periods where the animal was mobile (velocity > 5 cm s^−1^), including also distributions for shuffle-controlled firing rates (red, *N* = 880 neurons from 9 datasets from 4 animals). **h**, As for **g**, but for periods where the animal was stationary (velocity < 5 cm s^−1^). Datasets as for **g**. **i**, As for **g**, but for all neurons in L4 (*N* = 298 neurons from 9 datasets from 3 animals). **j**, As for **h**, but for the neurons in L4.

We next compared neuronal activity from populations of either L4 or L6 neurons while mice were freely exploring the linear track in either lit or dark conditions^28,29^ (Fig. 5a–d, Supplementary Video 5). Across L6 populations, neuronal activity ranged from active neurons in lit conditions becoming almost silent upon transition to dark to neurons that had sparse activity during lit conditions becoming active in darkened conditions (Fig. 5b). By contrast, L4 population activity was elevated in the light and sparse in the dark, but we observed few if any neurons with elevated activity in darkness (Fig. 5c,d). The animals were similarly active in both lit and dark conditions. For datasets recording activity in populations in either L4 or L6, average animal velocity in lit and dark epochs were not significantly different (mean ± s.d., L4 datasets lit, 9.2 ± 1.2 cm s^−1^, dark 8.9 ± 0.9 cm s^−1^, *N* = 3 animals, *P* = 0.60; L6 datasets, lit 9.2 ± 0.8 cm s^−1^, dark 9.0 ± 0.9 cm s^−1^, *N* = 4 animals, *P* = 0.69, Wilcoxon rank sum test in both cases). Using a previously published algorithm^30^ we inferred neuronal spiking from Ca^2+^-fluorescence traces (Fig. 5b and Supplementary Fig. 12) and calculated a light preference index (LPI; Supplementary Text) for each neuron. Neurons within local populations in both L4 and L6 showed no systematic spatial distribution or spatial clustering of neurons on the basis of their LPI in either layer (Fig. 5e,f). As neuronal activity in V1 can be modulated differentially (either enhanced or suppressed) by vestibular generated signals in lit or dark environments^28^, we further split our datasets into epochs where the animal was either mobile or stationary within light and dark conditions, and consequently with greater or lesser vestibular generated signal. For L6, whether the animal was mobile or stationary, we observed significant populations of neurons whose activity was strongly enhanced either in light (LPI around 1) or dark (LPI around −1) (Fig. 5g,h; observed distribution of LPIs versus LPIs from shuffled data, mobile/stationary, *P* = 4.3 × 10^−36^/*P* = 1.3 × 10^−49^, two-sample Kolmogorov–Smirnov tests, *N* = 9 datasets from 4 animals). In addition, consistent with modulation of neuronal activity by motion-driven vestibular input^28^, a significantly greater proportion of the recorded neurons fell into the extreme LPI categories (that is 1 or −1) when the animal was moving compared to when stationary (Fig. 5g,h; L6 LPI distribution stationary versus mobile, *P* = 1.9 × 10^−5^, two-sample Kolmogorov–Smirnov test, *N* = 9 datasets from 4 animals; Supplementary Fig. 13). For L4 populations, we observed neurons with activity enhanced by light both when the animal was mobile and stationary (Fig. 5i,j; observed distribution of LPIs versus LPIs from shuffled data, mobile/stationary, *P* = 1.5 × 10^−26^/*P* = 2.4 × 10^−36^, two-sample Kolmogorov–Smirnov tests, *N* = 9 datasets from 3 animals), and there was also a significant influence of motion on the distributions of LPIs observed (Fig. 5i,j; L4 LPI distribution stationary versus mobile, *P* = 0.003, two-sample Kolmogorov–Smirnov test, *N* = 9 datasets from 3 animals; Supplementary Fig. 13), again consistent with neuronal modulation by motion-driven vestibular input^28^. Compared to the L6 populations, this motion-induced increase in activity was more pronounced for neurons with a preference for light.

## Discussion

Here we present a 2-g miniature head-mounted three-photon microscope^9^ with remote focusing, suitable for imaging neuronal activity from populations located in any cortical layer in freely moving mice. The microscope also contains an on-board two-channel detector system that is robust to ambient light, which together with a scanner-synchronized arena lighting system enabled imaging of neuronal activity in a fully lit behavioral arena.

Three-photon excitation substantially extends the depth^31–33^ that neuronal population activity can be imaged in a freely moving animal^9^. We built a lightweight, remotely operated, mechanical focusing mechanism^15^ that enabled the imaging plane to be moved over larger distances through the cortex while the animal roamed freely, without the need to interfere with the animal’s behavior. The best axial resolution range could be positioned at any cortical depth, allowing movement between different neuronal populations located within a single layer or located in different layers, while still retaining high axial resolution and single-cell resolution, albeit without the advantage of simultaneous volumetric imaging^2,24,34^. Providing defocus for the remote focuing mechanism with an ETL^18^ provided faster and more precise control of the shifts in depth of the imaging plane^2,4^, with the penalty of an increase in microscope weight and reduced *z*-range. ETLs also allow imaging of multiple imaging planes by rapid switching of imaging plane depth, though with reduced imaging rate for the individual imaging planes^2,4^. The robust and lightweight SiPMs improved the signal-to-noise ratio of the image, reduced the cable bundle stiffness, allowed imaging to be performed in a lit behavioral arena, and provided rich structural information via the THG signal^16,17^. Our three-photon head-mounted microscope opens possibilities to image activity from neuronal populations located in any cortical layer during vision-based behaviors in lighting conditions simulating a far wider array of natural conditions^35^. Together this opens up the possibility of exploring the link between neuronal activity from all cortical layers and natural behavior.

## Methods

### Animals and surgical procedures

All animal experiments were conducted in accordance with the animal welfare guidelines of the Max Planck Society and with animal experimentation approval granted by the Landesamt für Natur, Umwelt und Verbraucherschutz Nordrhein-Westfalen, Germany.

Mice were housed in an SPF temperature-controlled (21 ± 1°) and humidity-controlled (>45%) facility on a 12 h light–dark cycle with food and water available ad libitum. Mice were group-housed until surgery and singly housed afterwards. Experiments were performed with twelve layer 4/layer 6-Cre mice (see below), six for imaging experiments with the miniature 3-photon microscope and six for comparison of behavioral statistics with and without the microscope. For imaging experiments, the mice (2 males, 5 females) were 8–98 weeks old (average of 23.5 weeks) and weighed 20–32 g (average of 23.0 g) at the time of the virus injection. For the behavioral comparison, the mice (6 females) were 20–58 weeks old (average 37 weeks) and weighed 27–32 g (average 30 g) at the time of the experiments.

The mice (*Mus musculus*) used in this study resulted from crossing heterozygous animals from two Cre-expressing lines, Ntsr1-Cre mice and Scnn1a-Cre mice. *Ntsr1* (neurotensin receptor 1)-Cre mice (B6.FVB(Cg)-Tg(Ntsr1-cre)Gn220Gsat/Mmcd) were obtained from the Mutant Mouse Resource and Research Center (MMRRC, #030648-UCD) and donated by N. Heintz (The Rockefeller University, GENSAT) and C. Gerfen (National Institutes of Health, National Institute of Mental Health), with Cre recombinase predominantly expressed in layer 6 of the cortex^21^. *Scnn1a* (sodium channel, nonvoltage-gated 1 alpha)*-Cre* mice (Tg(Scnn1a-cre)3AibsTg(Scnn1a-cre)3Aibs) were obtained from the Jackson Laboratory (#009613) and donated by E. Lein (Allen Institute for Brain Science) and T. Zwingman (Allen Institute for Brain Science) with Cre expression in cortex layer 4^20^. The Cre animals were maintained in a heterozygous state with C57BL/6J background. Expression patterns of these transgenic Cre lines have been described in the original papers cited.

The presence of the individual transgenes was confirmed by genotyping PCR with layer-specific primers. Primers for Snn1a-Cre animal specific PCR were 5′-AAAGAGAAGCGGGAGTCAG-3′ and 5′-GACCGGCAAACGGACAGAAG-3′. Primers for genotyping of Ntsr1-Cre animals were 5′-TCCCAGGATCTCCTGGATAG-3′ and 5′-GACCGGCAAACGGACAGAAG-3′ (forward and reverse primer, respectively).

For virus injections AAV1/2.hSyn.FLEX.jGCaMP7f was purchased from Addgene, the plasmid pGP-AAV-syn-FLEX-jGCaMP7f-WPRE was a gift from D. Kim and the GENIE Project (HHMI Janelia Farm Research Campus; Addgene plasmid #104492)^36^.

#### Genotyping PCR

Genomic DNA was isolated from ear biopsies using lysis buffer (10 mM Tris/HCl pH 8.0; 100 mM EDTA pH 8.0; 0.5% SDS) containing 1 mg ml^−1^ Proteinase K with subsequent isoropanol precipitation and ethanol wash steps. PCR was performed using DreamTaq Master Mix (ThermoFisher) and construct-specific primers.

#### Surgical procedures and imaging in freely moving animals

The procedure for labeling neurons with jGCaMP7f is provided in Supplementary Text.

All surgical instruments and solutions used were autoclaved before the procedures described below. Three to five weeks after the surgery to label neurons with jGCaMP7f, animals were anesthetized with a three-component anesthetic cocktail (3K) consisting of fentanyl (50 µg kg^−1^, Hameln pharma plus), midazolam (5 mg kg^−1^, Hameln pharma plus) and medetomidine (0.5 mg kg^−1^, Zoetis), and body temperature was maintained at 37–37.5 °C. Animal status and depth of anesthesia monitoring procedures were as described in the section on labeling neurons with jGCaMP7f in Supplementary Text. Anesthesia was maintained with supplementary doses of 30–80% of the initial dose of 3K solution. The hair on the dorsal aspect of the head was removed and the skin cleaned with 70% ethanol. A midline incision in the skin over the parietal bones was made, the skin retracted and galea removed to expose the parietal bones, including the site of the previous burrhole. The exposed bone was then cleaned with hydrogen peroxide solution (3% by volume in sterile saline) and thoroughly washed with sterile saline. The bone was then mechanically roughened before application of a layer of dental adhesive (Optibond). The custom-made headplate (Supplementary Fig. 14, Supplementary Video 6 and Supplementary Text) was then fixed to the skull over the Optibond layer with dental composite (Charisma, Kulzer GmbH). The central aperture was placed such that the burrhole from the previous surgery was located approximately centrally in the medial–lateral axis and near the anterior edge of the aperture. The skin incision was then closed firmly around the headplate using 5/0 vicryl sutures (Ethicon). A circular craniotomy with a diameter of approximately 3 mm was then opened in the center of the headplate aperture, including at the anterior margin of the site of the previous craniotomy. The dura was then removed and the cranial window closed using a pre-formed plug and coverslip (circular, 5 mm diameter, 100 µm thickness, CS-5R-0, Warner Instruments; plug custom pre-formed as a 300 or 400 µm tall cylinder of KwikSil silicone in the center of the circular coverslip after the description in ref. ^37^). The plug was designed to approximately match the gap between the surface on which the coverslip rested and the bottom of the bone, including the thickness of the headplate and the adhesives used to fix it to the skull. During placement of the coverslip and plug every effort was made to avoid application of excessive pressure on the cortical surface, to not compromise blood flow through the cortical blood vessels. Subjectively, increased pressure of the plug on the cortical surface did not improve imaging stability in the deeper cortical layers. The 3PE employed by this microscope has been shown to allow imaging through the dura^38^, though we removed the dura here to ensure the best possible image quality in the deep cortical layers where the effects of light scattering are higher. Employing a coverslip only with no silicone plug may also be possible^1^, though we elected to use a plug to minimize the potential for damage to the cortical surface and blood vessels should the cortical surface come into contact with the edges of the region where the bone had been removed.

The animal was then transferred to the miniature microscope, which was mounted on a navigation stage for locating an appropriate position for imaging within the cranial window. The navigation stage consisted of a micromanipulator (MP-285, Sutter Instruments) to which the microscope was mounted using a custom-made mount. The mount included two angular kinetic mounts (GN05/M and GN1/M, Thor Labs) allowing adjustment of tilt with respect to the coverslip and cortical surface. Once a target population of neurons had been located, the intermediate attachment plate (already mounted to the miniature microscope) was attached to the headplate with dental composite (Charisma Flow, Kulzer GmbH), the microscope removed and the animal administered a cocktail of antagonists to the anesthetic drugs (anti-3K) consisting of naloxone (11.2 mg kg^−1^, Ratiopharm), flumazenil (0.5 mg kg^−1^, Hikma, Amman Jordan) and atipamezole (0.75 mg kg^−1^, Orion Pharma).

Freely moving experiments were subsequently conducted from 2 to 10 days after opening the cranial window and positioning the microscope. At the commencement of each recording session, the animal was taken from its home cage, the head gently restrained by holding the handle on the back of the headplate, and the microscope placed onto the intermediate attachment plate. Placement of the microscope was rapid, taking in the order of a few seconds. After microscope placement, the animal was placed onto the linear track and allowed to explore. Candidate populations of neurons were located by navigating with the remote *z*-drive mechanism, and then data were acquired. Frame rate and acquisition parameters were 273 × 280 pixels per frame, 10.6 frames per second. At the end of a recording session, the animal was again gently removed from the linear track, the screw on the microscope mounting plate unfastened and the microscope removed. This mechanism allowed the microscope, and particularly the objective that projected through the microscope mounting plate and intermediate attachment plate and made direct separation of the plates difficult, to be lifted vertically away from the headplate without having to apply the force required to separate the magnets. With the microscope removed, the magnets holding the microscope mounting plate and intermediate attachment plate could be separated with the animal’s head restrained and supported again using the tab on the headplate, and the intermediate attachment plate removed. The central aperture of the intermediate attachment plate was then closed using a cap that was also fitted with matching magnets, after which the animal returned to its home cage.

A description of the linear track and the methods for tracking animal position and head orientation, as well as histological methods are provided in Supplementary Text.

#### Behavioral comparison

For comparison of the characteristics of animal movement, naive mice that had not undergone any previous procedures were placed on the linear track described above and data were acquired for 20 min while the animals freely and spontaneously explored the track. For tracking animals without any LED markers, we installed a camera (acA1300–200um, Basler AG) above the center of the track, with the optical axis pointing vertically down, such that the track surface was parallel to the image sensor. For comparison of the animals’ behavioral characteristics while carrying the microscope or just the position-tracking struts, either the miniature microscope or a head-mount consisting of only the position-tracking struts were placed on the intermediate attachment plate, and the animal allowed to spontaneously explore the track for a session of 20 min duration. Sessions with the microscope and with the position-tracking strut-only head-mount were conducted with each animal to allow direct comparison of the animal’s behavior, with an interval of three days elapsing between the two behavioral sessions for each animal. At the end of a session, close-up images of the animals were acquired with cameras calibrated as described in Supplementary Text such that the tracking LEDs as well as the animal’s eyes and nose were simultaneously visible. These images were subsequently used to precisely measure the position of the eyes and nose relative to the tracking LEDs so that the true pitch of the animal’s head could be determined from the tracking data for comparison between the microscope and tracking struts-only datasets (that is, to account for differences in the orientation of the position-tracking struts between the two different head-mounts).

### Microscope and optical setup

#### Excitation laser setup

For excitation, we used either a non-collinear optical parametric amplifier (NOPA), pumped by a Ytterbium fiber laser (Spirit-NOPA, Spectra Physics) that produced laser pulses with a maximum average power of 3 W (3 µJ at 1 MHz) or an optical parametric chirped pulse amplifier (OPCPA), pumped by a Ytterbium fiber laser (white dwarf dual, Class 5 photonics GmbH) that produced pulses with a maximum average power of 5 W (1.25 µJ at 4 MHz). In both cases, the center wavelength was 1,300 nm, and pulses were sub-50 fs at the output of the laser. We used a half-wave plate (AHWP05M-1600, Thorlabs) mounted on a stepper motor-controlled rotator (G065118000, Qioptiq) with a polarization beam splitter (PBS104, Thorlabs) to control beam intensity. To compensate dispersion introduced by the fiber we used a two-prism sequence with additional bulk silicon described previously^9^. In brief, to compensate third-order dispersion, a double-path two-prism sequence compressor with Brewster-angle silicon prisms at 32° apex angle (4155T724, Korth Kristalle Gmbh) was used. We used a custom-designed second prism with a larger base of 56 mm to increase the compensation range. Additional anomalous group-velocity dispersion generated by the prism sequence was compensated using additional bulk silicon. To pre-compensate the dispersion of the 1.5 m of fiber used in the experiments in the current study we used an inter-prism distance of 35 cm and an additional 10 cm of silicon. The beam was coupled to the previously designed custom hollow-core fiber^9^ with a 10 mm focal length achromatic lens (AC050-010-C-ML, Thorlabs). A half-wave plate (AHWP05M-1600, Thorlabs) was used to control the linear polarization orientation of the laser with respect to the fiber structure and a detuned 1:1 relay telescope enabled optimization of the coupling efficiency of the fiber.

#### Miniature optics design and optimization

We used OpticStudio 14.2 (Zemax Europe) to design the full optical system for the three-photon miniature microscope. The system was composed of a collimation lens, scanning optics (scan lens system and tube lens) and an imaging objective lens (Supplementary Fig. 15). The objective lens was designed first, and then all the other parts were optimized as part of the full system with objective lens parameters kept constant. The primary design parameters for the objective lens were: large working distance (>1.5 mm), high collection NA (>0.9), excitation NA up to 0.6 and large FOV (>400 µm, at 0.4 excitation NA). As 3PE requires short pulses, chromatic aberrations constitute a particular concern for microscopes employing it. To account for it, three wavelengths were used with equal weight for the simulations (1,250 nm, 1,300 nm, 1,350 nm). The polychromatic Strehl ratio was used as the main quantification of the optical performance.

Compensating field curvature requires complicated, large and heavy objective lenses^39^. A fully uncompensated field curvature led to about 10 µm axial distance between the axial focal point and the outer part of the FOV. This parameter was left floating and uncompensated to keep the objective lens as small as possible, and because such a field curvature was not deemed to be an obstacle to measure neuronal activity in the intact brain, as 10 µm distributed over 200 µm of half FOV produces a small *z*-slope in relation to a *z*-resolution > 10 µm.

To keep the weight and the size of the system minimal, several approaches were used during the design process. First, the EFL of the objective was decreased compared to the previous design^9^ (1.8 mm from 3 mm). This resulted in much smaller beam diameters employed to achieve the same NA (3.6 mm compared to 6 mm for an NA of 0.9) and allowed the use of much smaller and thinner lenses. The focal lengths of the scan and tube lenses were also minimized to shorten the total beam path. The limitation to this approach is the proportionally larger scanning angles (compared to beam diameter decrease) required to scan the same FOV. Focal lengths were systematically explored and the best balance (achieving minimal weight and size) was selected for the design.

The same guidelines were used to design scanning optics and the collimation lens. A 2.0 mm diameter MEMS scanner (A7M20.1-2000AU-LCC20-C2TP, Mirrorcle) was selected, featuring ±4.5 mechanical degrees of scanning range. To minimize the manufacturing costs the tube and scan lenses were constrained to contain the same achromat. The tube lens is the achromat itself and the scan lens is composed of the achromat and a plano-convex (PCX) lens of either 15, 12 or 9 mm EFL (3 mm diameter, EFL of 15 mm, 12 mm and 9 mm, PCX lenses, NIR II, Edmund Optics) to provide a range of magnifications. The focal length of the achromat was chosen to be 6.68 mm, to provide 0.4 excitation NA with the scan lens containing a PCX of 15 mm EFL. In the case of 12 or 9 mm EFL PCX lens the excitation NA was 0.43 and 0.48 respectively.

Additional constraints were provided by the requirement for imaging under defocus conditions to implement remote focusing (Supplementary Fig. 15). To minimize the system complexity and size, an unfolded beam path was chosen with the defocus provided by a varying distance between the hollow-core fiber (HCF) and the collimation lens. The distance from the collimation lens to the MEMS scanner was the EFL of the collimation lens to provide constant excitation NA over the *z*-range. The combination of the collimation lens EFL and the NA of the HCF with the magnification of the scan-tube lens system give the final excitation NA. The design parameters of the *z*-drive were: a *z*-range of >600 µm (covering a substantial part of the mouse cortex in depth) with >0.4 excitation NA and >400 µm FOV.

Achieving an NA of 0.4 requires a beam diameter of 1.15 mm at the back focal plane (BFP) of the objective. A FOV of ±200 µm requires a scan angle of arctan(±0.2/1.8) = ±6.34° at the BFP. Considering the deflection properties of the MEMS scanner a magnification of 2 × 4.5°/6.34° = 1.42 is required in the scan-tube lens system. With a magnification of 1.42, to achieve a beam diameter of 1.15 mm at the BFP of the objective, a 1.15 mm/1.42 = 0.81 mm beam diameter is needed at the MEMS, which, provided an estimate of 0.09 NA of the HCF, requires a collimation lens of 4.5 mm EFL. We used a combination of two EFL 9.0, PCX lenses (#67-443, Edmund Optics) to decrease aberrations owing to defocus in the collimation lens, resulting in an EFL of 4.5 mm. The achromat used as tube and scan lens in combination with a PCX lens with an EFL of 15 mm yielded a magnification of 1.42 of the scan-tube lens system. All previous values were kept constant during the optimization of the design. Resulting systems had an excitation NA of 0.41, 0.44 or 0.48 (using PCX with an EFL 15, 12 or 9 mm, respectively, in scan lens group). To further increase the excitation NA, it is possible to change the collimation lens to two PCX with 12 mm EFL. With a scan lens group utilizing a PCX with 12 mm EFL it results in a total excitation NA of 0.58. This system was assembled and characterized and the results are shown in Supplementary Fig. 3.

A dichroic mirror (T875spxrxt, AHF Analysentechnik AG) was used to separate the excitation beam path and the emitted fluorescence. A condenser lens with an EFL of 5 mm was used (Throl GmbH). To produce a high-performance miniature infrared filter, we used an existing filter with an optical density of 7 in the range 900–1,600 nm (T875spxrxt-1800-UF1, Chroma Technology Corp), which was cut in circular pieces 4 mm in diameter. The resulting pieces were separated in two groups and ground down to a thickness of 0.3 mm starting from either side of the filter. Pairs were cemented together (one piece from each group) to obtain a 4 mm diameter filter with the original performance and thickness of 0.6 mm. A dichroic mirror (DMLP490R, Thorlabs) separated detected light into two channels. This mirror was cut into rectangular pieces of 2 × 3 mm and ground down to 0.3 mm thickness. We used square, 1.3 mm side, silicon photomultipliers (S13360-1375PE, Hamamatsu) as detectors on-board of the microscope (see below). The resulting system is shown in Supplementary Fig. 9.

For the experiments with the ETL we used an electrowetting lens (A-25H1-D0, Corning), with the compatible driver (USB-M Flexiboard, Corning) and a relay lens system composed of 4 lenses, 2 with EFL 12 mm and 2 with EFL 9 mm (#67-444 and #67-443, Edmund Optics) as shown in Supplementary Fig. 4.

#### Microscope and *z*-drive manufacturing

The structure of the microscope body, implants and lens mounts (Supplementary Fig. 16 and Supplementary Video 7), including objective, were designed with Inventor (Autodesk GmbH). A 3D printer (Form3, Formlabs) was used to produce the microscope body in black resin V4 (Formlabs) and the headplate in a biocompatible, glass-filled resin (Temporary CB). All the optics and the objective frame, made of titanium, were supplied by Throl GmbH.

The HCF was passed through a jacket (FT900Y, Thorlabs). The end of the jacket from the laser-to-fiber coupling side was glued in a plate for a 30 mm cage system (Thorlabs). A syringe was then glued on this plate, centered and aligned along the axis of the cage. To prevent the fiber from buckling during sliding, the bare fiber was passed through that syringe first, then through a smaller syringe with an outer diameter that matched the inner diameter of the first syringe, such that the second syringe could slide inside the first (Supplementary Fig. 17 and Supplementary Video 8). The bare fiber was glued inside the second syringe, so displacement of this syringe inside the cage system was displacing the bare fiber inside its jacket. The jacket was rigidly attached to the microscope body, so the displacement of the fiber inside the jacket resulted in variation of the distance of the fiber tip to the collimation lens from <1 mm to >14 mm. The actuation was performed using a stepper motor (ZST213, Thorlabs). On the microscope side, the fiber tip was glued inside a ceramic ferrule of 2.5 mm diameter (CF128-10, Thorlabs) that slid inside a corresponding structure on the microscope (Supplementary Fig. 17), which was 3D printed with the diameter adjusted with a reamer to minimize sliding friction.

#### Fluorescence detection, control electronics and software

Scanimage 5 (Vidrio Technologies) with custom FPGA code featuring the triggered acquisition was used to control the microscope. The MEMS scanner was operated in the resonance mode in the fast axis at 1.5 kHz period providing 3,000 lines per second using bi-directional scanning. This, combined with a 1 MHz excitation laser pulse rate and 82% of imaging fill fraction, resulted in a resolution of about 273 pixels × 280 lines at 10.6 Hz. The FOV during the activity data collection was a square of up to 300 × 300 µm². The driving signals from Scanimage software were sent to the MEMS driver (BDQ_PicoAmp_4.6, Mirrorcle).

#### Measured and theoretical optical resolution

The theoretical optical resolution of the microscope, assuming perfect alignment of the optical components, is independent of the defocus-based focuing mechanism, and consequently should be constant with varying focus depth. In practice, the slightly imperfect alignment of the optical elements in the finally assembled microscope result in a dependence of the optical resolution on the extent of defocus, and consequently variable optical resolution with focus depth. We have therefore quantified and reported in figures and text the measured optical resolution over the available range of focus depths for both the extended *z*-range and high-resolution configurations of the microscope optics. Optical resolution was measured using a sample of 500 nm fluorescent beads (F8813, Thermo Fischer Scientific), using image stacks with 1 µm *z*-step. The theoretical optical resolution of the system was calculated using vectorial diffraction theory, and assuming an NA for the hollow-core fiber of 0.09, on the basis of the mode field diameter. The varying alignment results in the system NA being variable of the focus position, causing deviations from this theoretical value. For this reason, to compare measured and theoretical optical resolution we have used the average measured axial and lateral optical resolution over the focus range. Theoretical optical resolution for extended *z*-range, axial 10.5 µm, lateral 0.98 µm, average measured optical resolution axial 13.8 ± 3.8 µm, lateral 1.18 ± 0.15 µm (both mean ± s.d.). For the enhanced resolution version, theoretical optical resolution, axial 6.7 µm, lateral 0.79 µm, average measured optical resolution axial 10.1 ± 3.9 µm, lateral 1.11 ± 0.2 µm (both mean ± s.d.).

#### Lens combinations required for the extended-focus and high-resolution configurations

The commercial lenses contained in the excitation path of the extended z-range version are:

two EFL 9.0, PCX lenses (#67-443, Edmund Optics) for the collimation lens system and one EFL 9.0, PCX lense (#67-443, Edmund Optics) in the scan lens system. For the enhanced resolution version, we used two EFL 12.0, PCX lenses (#67-444, Edmund Optics) for the collimation lens system and one EFL 12.0, PCX lenses (#67-444, Edmund Optics) in the scan lens system.

#### Environment light

The environment of the track was homogeneously illuminated using six 24 V RGBW LED strips of 125 cm length with 910 lm m^−1^ and eight 12 V white LED strips of 125 cm length with 700 lm m^−1^ (both LED strips from PowerLED), arranged equidistantly in a patch of 125 × 60 cm^2^ at a distance of 150 cm above the track.

The strips were switched on and off in synchrony with the line signal of the microscope using a custom circuit based on TIP120 Darlington transistors.

A description of the procedure for comparison of SiPM and POF detections systems, the protocol for volumetric imaging and a full description of all data analysis methods are provided in Supplementary Text.

### Reporting summary

Further information on research design is available in the Nature Portfolio Reporting Summary linked to this article.

## Online content

Any methods, additional references, Nature Portfolio reporting summaries, source data, extended data, supplementary information, acknowledgements, peer review information; details of author contributions and competing interests; and statements of data and code availability are available at 10.1038/s41592-022-01688-9.

## Supplementary information

Supplementary Information Supplementary Figs 1–17 and Supplementary Text.

Reporting Summary

Supplementary Video 1 ***z*-stacks acquired using either the head-mounted** ***z*-drive or with the miniature microscope mounted on an external micromanipulator**. *z*-stacks from the brain surface to the white matter. All data has been acquired with the high *z*-range version of the microscope in primary visual cortex of an anesthetized mouse. Images are overlays of jGCaMP7f fluorescence (green) and the third-harmonic signal (THG signal, blue). From left to right: side projection of the stack acquired with the head-mounted *z*-drive; image stack acquired with the head-mounted *z*-drive; image stack acquired with the miniature microscope mounted on an external micromanipulator; side projection of the *z*-stack acquired using the external micromanipulator. Note that the first side projection has been scaled approximately by comparing visible structures with the conventional *z*-drive micromanipulator. Note also that the image stack acquired with the head-mounted *z*-drive was acquired dynamically while moving the fiber tip, and not with a step/acquire sequence, and consequently appears slightly smoothed compared to the image stack acquired on the external micromanipulator. Sudden increases in brightness in the THG signal occur as the fiber position moves through a range where the laser is temporarily focused on one of the internal optical elements of the microscope, generating a strong THG signal from the lens surface.

Supplementary Video 2 **Remote depth navigation between cortical layers 4 and 6**. All data has been acquired with the high *z*-range version of the microscope in visual cortex of a freely moving mouse. Fluorescence from jGCaMP7f shown in green, third-harmonic signal in blue. Imaging is shown at three times real speed. Left, imaging data shown with a ten-frame rolling average, while the head-mounted *z*-drive is used to change the imaging depth from layer 6 to the bottom of the cortex, with white matter visible in the blue channel, then up to layer 4. At layer 4 the FOV was also shifted by applying voltage offset to the MEMS scanner, adjusting the lateral position of the FOV. Right, visible spectrum overhead camera view, with outer front (white), outer left (red) and outer right (green) infrared LEDs positions labeled.

Supplementary Video 3 **Comparison of raw imaging data, a three-frame rolling average and denoised imaging data**. All data has been acquired with the high *z*-range version of the microscope in visual cortex of a freely moving mouse. Movies show green fluorescence from jGCaMP7f. Imaging is shown in real time. Top, from left to right: raw data, three-frame rolling average, imaging data processed with DeepCAD (Li et al. 2021). Bottom, fluorescence traces calculated from the imaging data in the same column from the neurons indicated by the colored circles in the movies. Data from a neuronal population in layer 6, data acquired with laser power 45 mW. (Li, X. *et al*. Reinforcing neuron extraction and spike inference in calcium imaging using deep self-supervised denoising. *Nat Methods* **18**, 1395-1400, doi:10.1038/s41592-021-01225-0 (2021)).

Supplementary Video 4 **Examples of raw image data and behavior in freely moving mice**. Raw fluorescence image data (left) and visible light overhead camera view (right) from example neuronal populations in layer 6 (upper) and layer 4 (lower) in two different sessions with two different animals. All data has been acquired with the high *z*-range version of the microscope in visual cortex of a freely moving mouse. Movies show green fluorescence from jGCaMP7f. Imaging is shown in real time. Laser power 27 and 18 mW, respectively, for fluorescence imaging in upper and lower raw data images.

Supplementary Video 5 **Imaging and mouse behavior with lit and dark environment**. All data has been acquired with the high *z*-range version of the microscope in visual cortex of a freely moving mouse. Movies show green fluorescence from jGCaMP7f. Imaging is shown in real time. Top left, imaging data processed with DeepCAD (Li, Zhang et al. 2021). Top right, visible spectrum overhead camera view with outer front (white), outer left (red) and outer right (green) infrared LED positions labeled. Bottom, fluorescence traces from the neurons indicated in the fluorescence images. Scale bar applies to all traces. Data from a neuronal population in layer 6, data acquired with laser power 38 mW.

Supplementary Video 6 **Animated CAD models showing the procedure for mounting the microscope on the head plate**. Animation illustrating the sequential steps during the positioning and mounting procedure and for an imaging experiment. The individual steps are shown in Supplementary Fig.8.

Supplementary Video 7 **Animated CAD models showing the process of assembling the microscope**. Animation of the assembly process as described in Supplementary Fig. 10.

Supplementary Video 8 **Animated CAD models showing the mechanical** ***z*-drive setup**. Animation of operation of the mechanical *z*-drive as shown in Supplementary Fig. 11.

## Data Availability

Source data and Zemax files are available at 10.5061/dryad.76hdr7t11.
