# Supplementary material for: A three-photon head-mounted microscope for imaging all layers of visual cortex in freely moving mice
Source: Nat Methods. 2022 Nov 28;20(4):610–6. doi: 10.1038/s41592-022-01688-9 (PMC10089923; doi:10.1038/s41592-022-01688-9)
Supplement: Supplementary file 1 — Supplementary Figs 1–17 and Supplementary Text. [file 41592_2022_1688_MOESM1_ESM.pdf]

nature methods

Article

https://doi.org/10.1038/s41592-022-01688-9

A three-photon head-mounted microscope for imaging all layers of visual cortex in freely moving mice

In the format provided by the authors and unedited

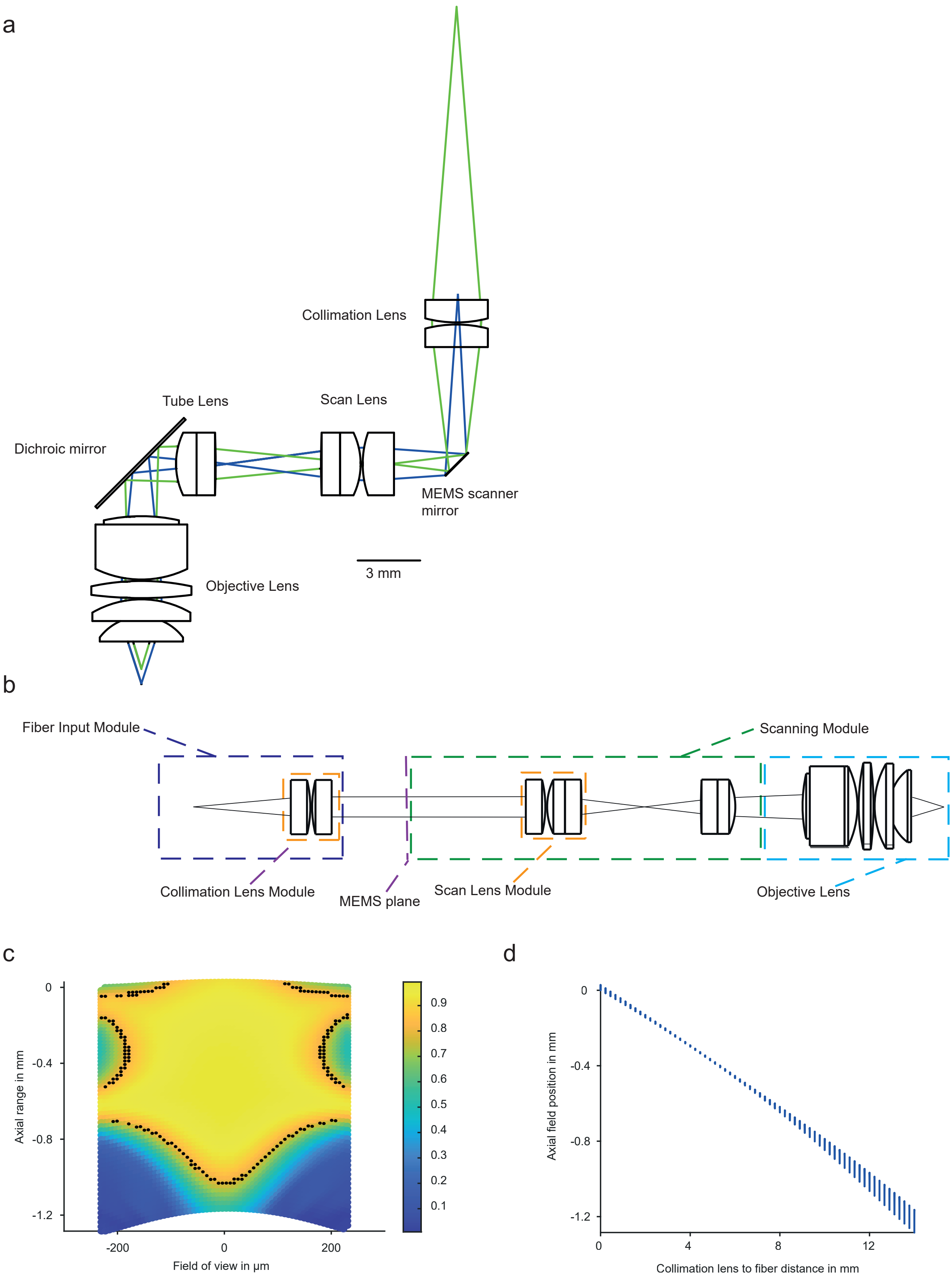

Supplementary Figure 1. Optical design and performance simulation in Zemax. a, optical layout of the microscope. Green and blue rays illustrate extreme position of the fiber tip leading to minimal and maximal working distance respectively. **b**, modular design illustrated on the linear optical layout of the microscope. **c**, simulated optical performance measured by polychromatic Strehl Ratio (SR, color-coded) over the field-of-view and the Z-range achievable by changing the fiber-collimation lens distance. Black dots delimit the diffraction limited imaging area (SR > 0.8). **d**, imaging depth relative to the maximum working distance as function of the fiber tip to collimation lens distance from Zemax simulation. Note that for each collimation lens to fiber tip distance there is a range of imaging depths because the objective is not corrected for flat field.

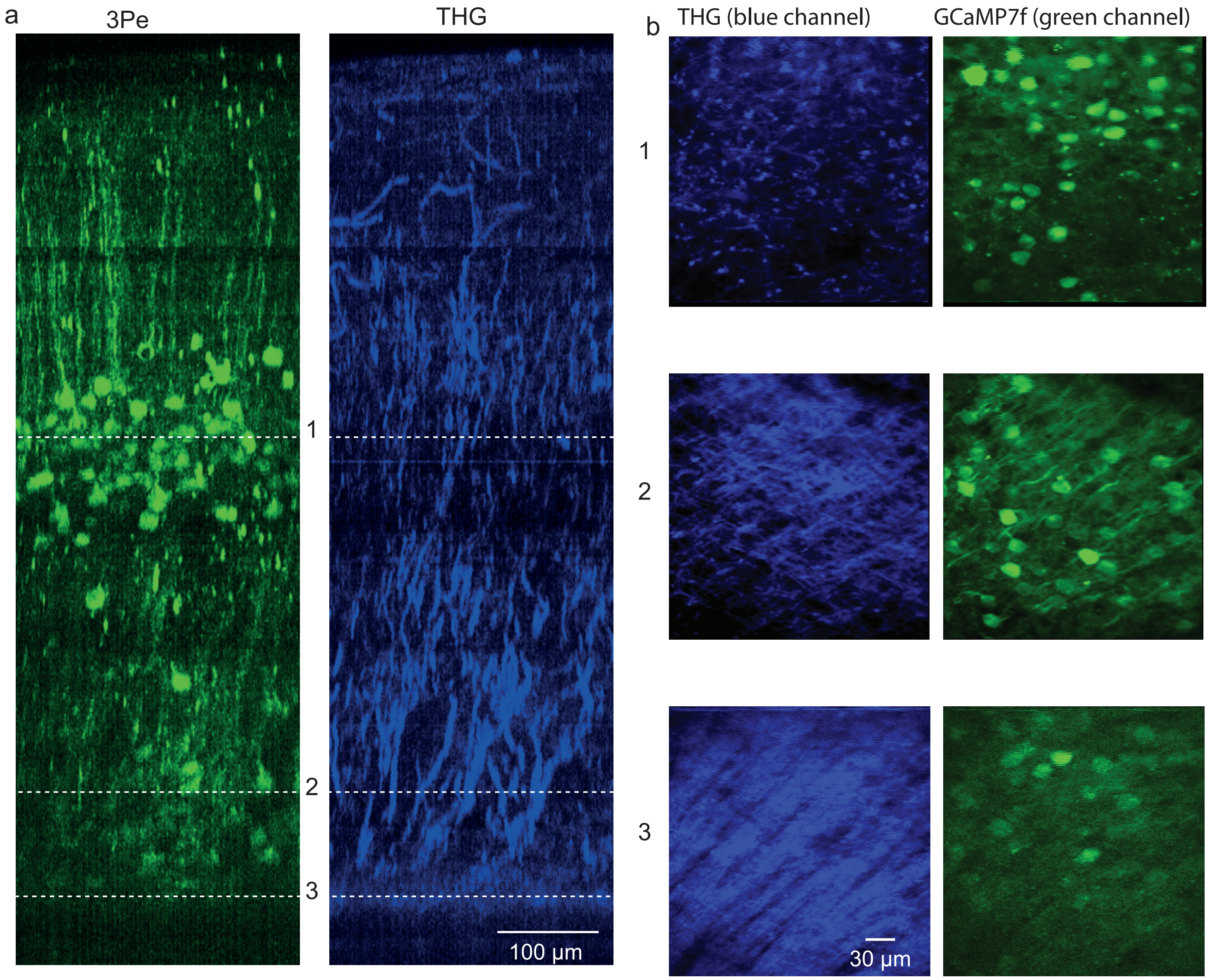

Supplementary Figure 2. Three-photon image stack from layer 1 to the corpus callosum. a, image stack of GCaMP7f-labelled neurons (left) and third harmonic generation signal (THG, right) acquired from an anesthetized mouse with the miniaturized microscope attached to an external micromanipulator. Changing focus depth was achieved using the external micromanipulator. Dashed lines indicate the depths at which the images in b were acquired. Scale bar in THG image applies to both images. **b**, individual images of GCaMP7f labelled neurons (right) and THG signal images (left) from the locations indicated in a. Scale bar in the number 3 THG signal image applies to all images in b. Data as in Fig. 1f &g.

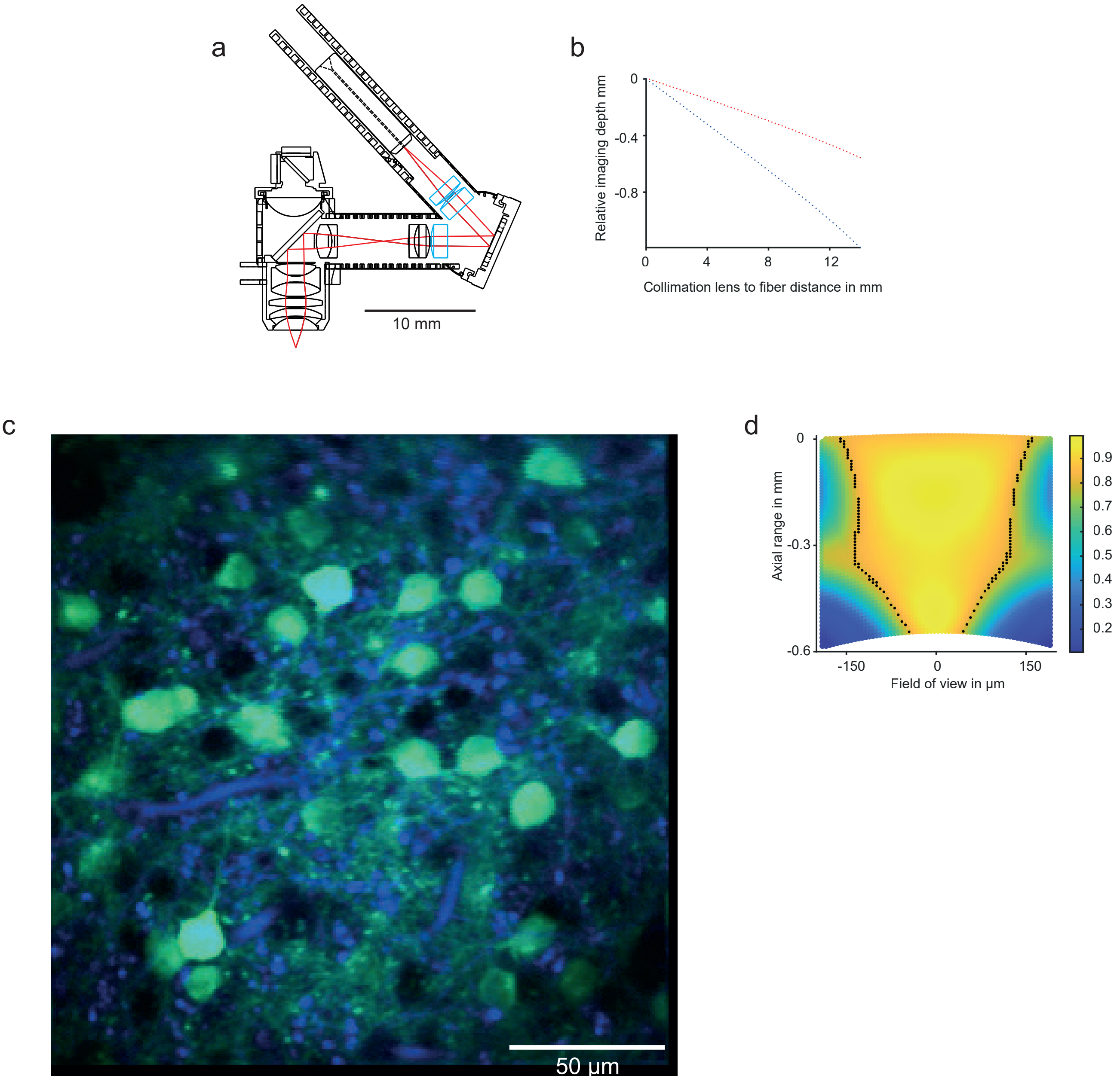

Supplementary Figure 3. The choice of commercial lenses determines the interplay between resolution, field-of-view and Z-range. a, microscope schematic showing the commercially available lenses which are exchanged to switch from the extended z-range to the enhanced-resolution version of the microscope. **b**, simulation of the z-range for the high z-range microscope presented in the main text (blue) and a high-resolution version with different set of COTS lenses (red). **c**, example field-of-view of the high-resolution version microscope in layer 4 of an anaesthetized mouse. Neurons recorded at 376 µm depth with laser power 8.8 mW. **d**, simulated optical performance for the high-resolution version measured by polychromatic Strehl Ratio (SR, color-coded) over the field-of-view and the z-range achievable by changing the fiber-collimation lens distance. Black dots delimit the diffraction limited imaging area (SR > 0.8).

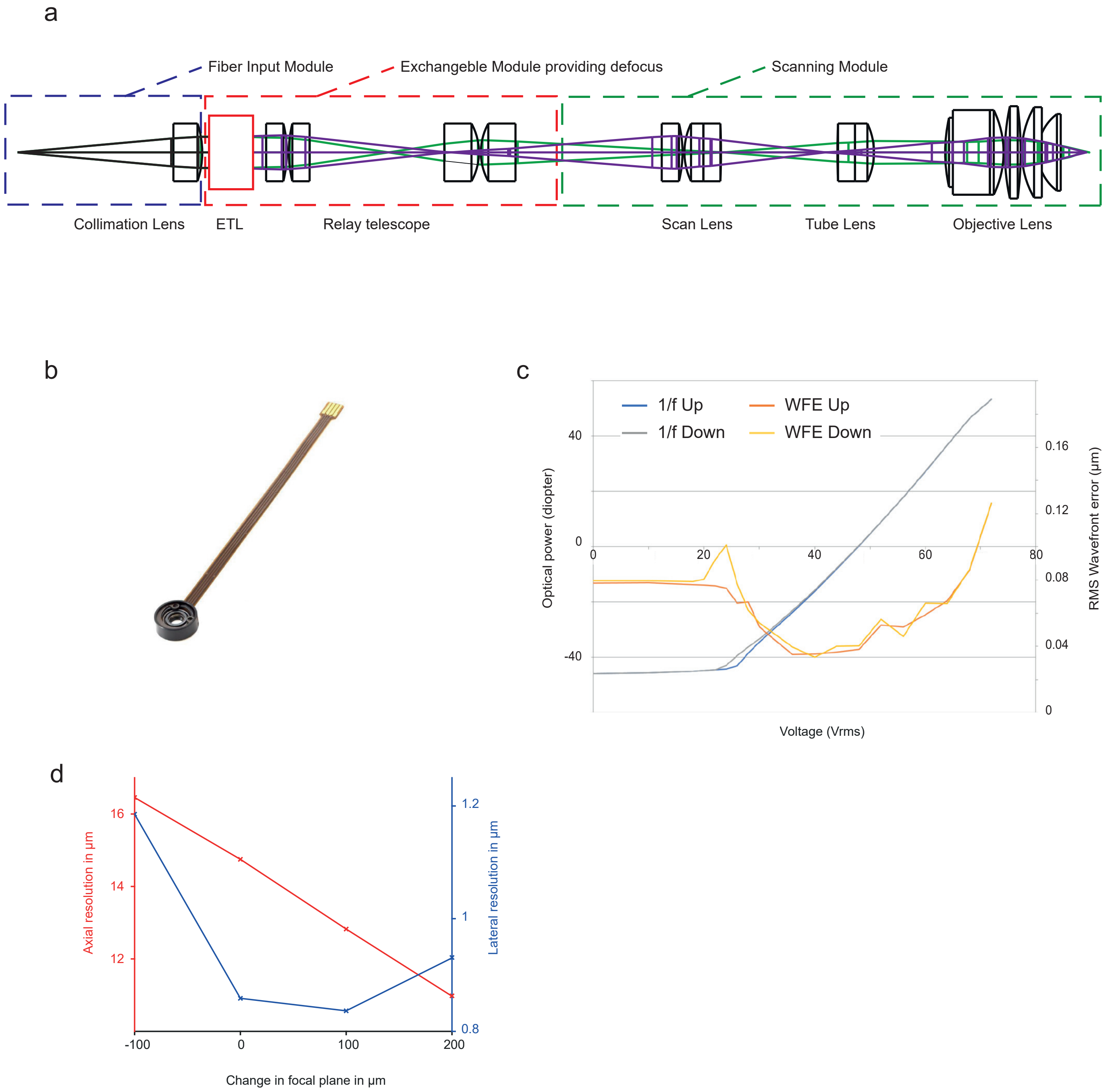

Supplementary Figure 4. Optical design of the microscope employing an electrically tunable lens for defocus. a, linear optical layout of the microscope using an electrically tunable lens. Violet and green rays show the beam in two extreme defocus positions giving minimal and maximal working distance respectively. **b**, photograph of a high dynamic range electro wetting electrically tunable lens. **c**, relation for the electrically tunable lens between applied voltage and optical power in diopters (blue, increasing voltage, gray, decreasing voltage) and RMS wavefront error in micron (orange, increasing voltage, yellow, decreasing voltage). b and c, reproduced from Varioptic lens documentation with permission from Corning. **d**, measured axial (red) and lateral (blue) resolutions of the electrically tunable lens based version of the microscope.

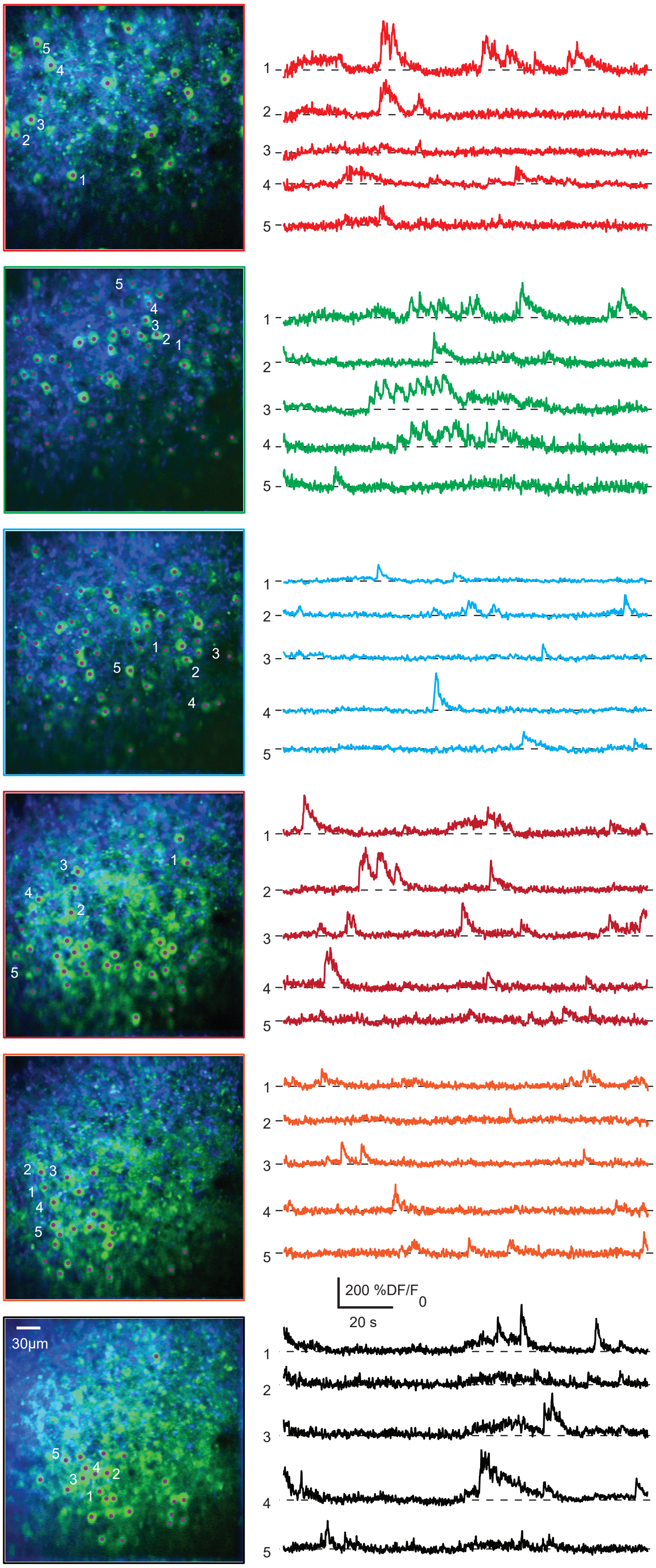

Supplementary Figure 5. Example of multiplane imaging. Overview images (left) and example calcium fluorescence traces (right) for 6 sequentially acquired image planes in layer 4 in a freely moving mouse. Same data as shown in Fig. 2e, but with focus planes separated for better visualization. Numbered neurons in overview image corresponds to the calcium fluorescence traces shown to the right. Scale for fluorescence traces applies to all traces, scale bar on bottom image applies to all overview images.

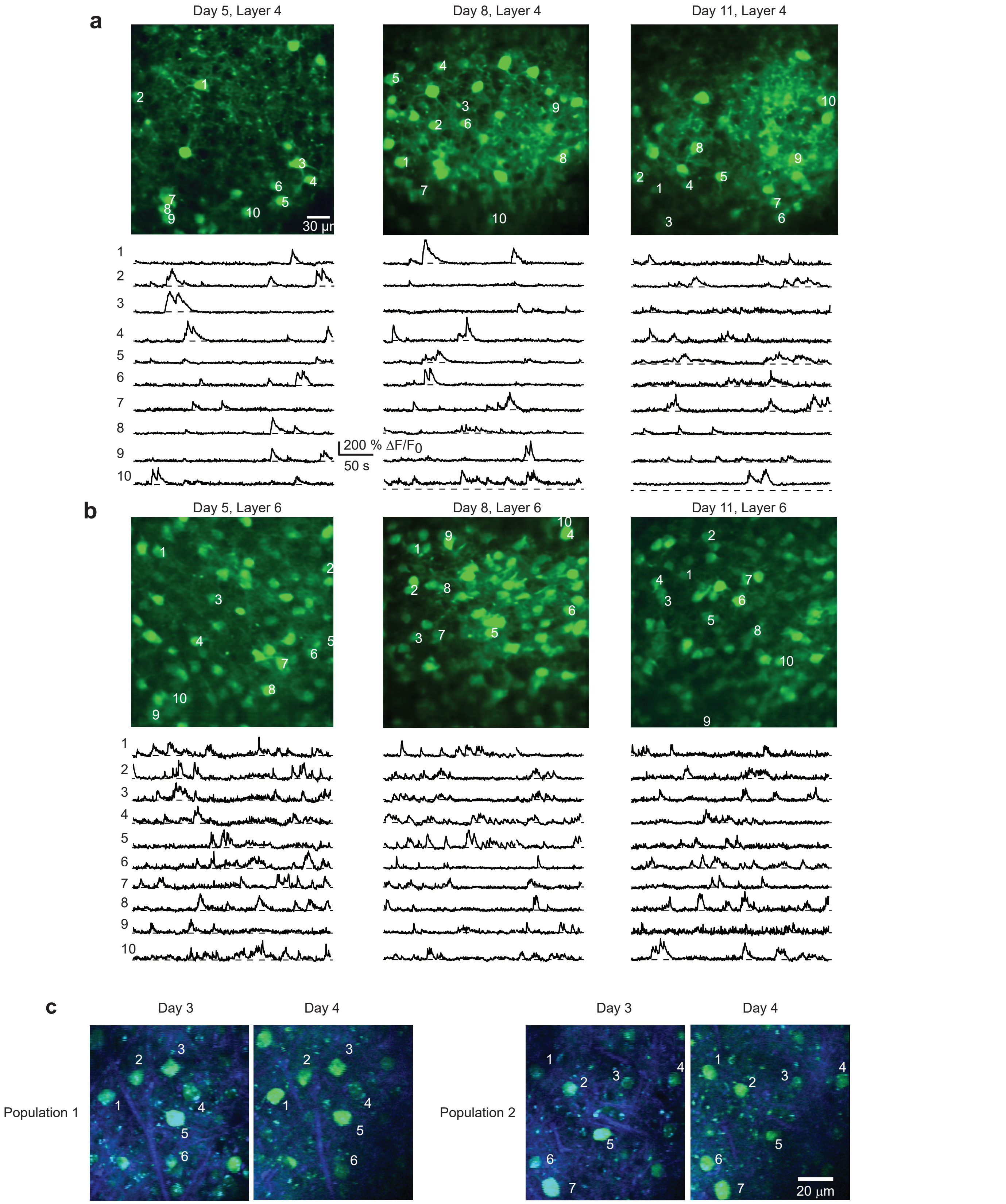

Supplementary Figure 6. Example of multi-day imaging using the miniature 3P microscope. a, upper panels show an imaging overview with ten example neuronal calcium-fluorescence traces from different fields-of-view on imaging day 5 (left column, 5 days after implantation of the cranial window), imaging day 8 (middle column) and imaging day 11 (right column), for fields-of-view in cortical layer 4. **b**, lower panels show an imaging overview with ten example neuronal calcium-fluorescence traces from different fields-of-view on imaging day 5 (left column, 5 days after implantation of the cranial window), imaging day 8 (middle column) and imaging day 11 (right column), for fields-of-view in layer 6. All data from one example animal. Scale bar in upper left overview image applies to all overview images, scale bars between left and middle upper neuronal calcium traces applies to all calcium traces. Numbering of neuronal calcium traces corresponds to numbering of neurons in the corresponding overview image. **c**, repeated imaging from the same neurons (green, GCaMP7s) in the same animal over 2 days (day 3 and 4) for two sperate L6 populations (populations 1 and 2).

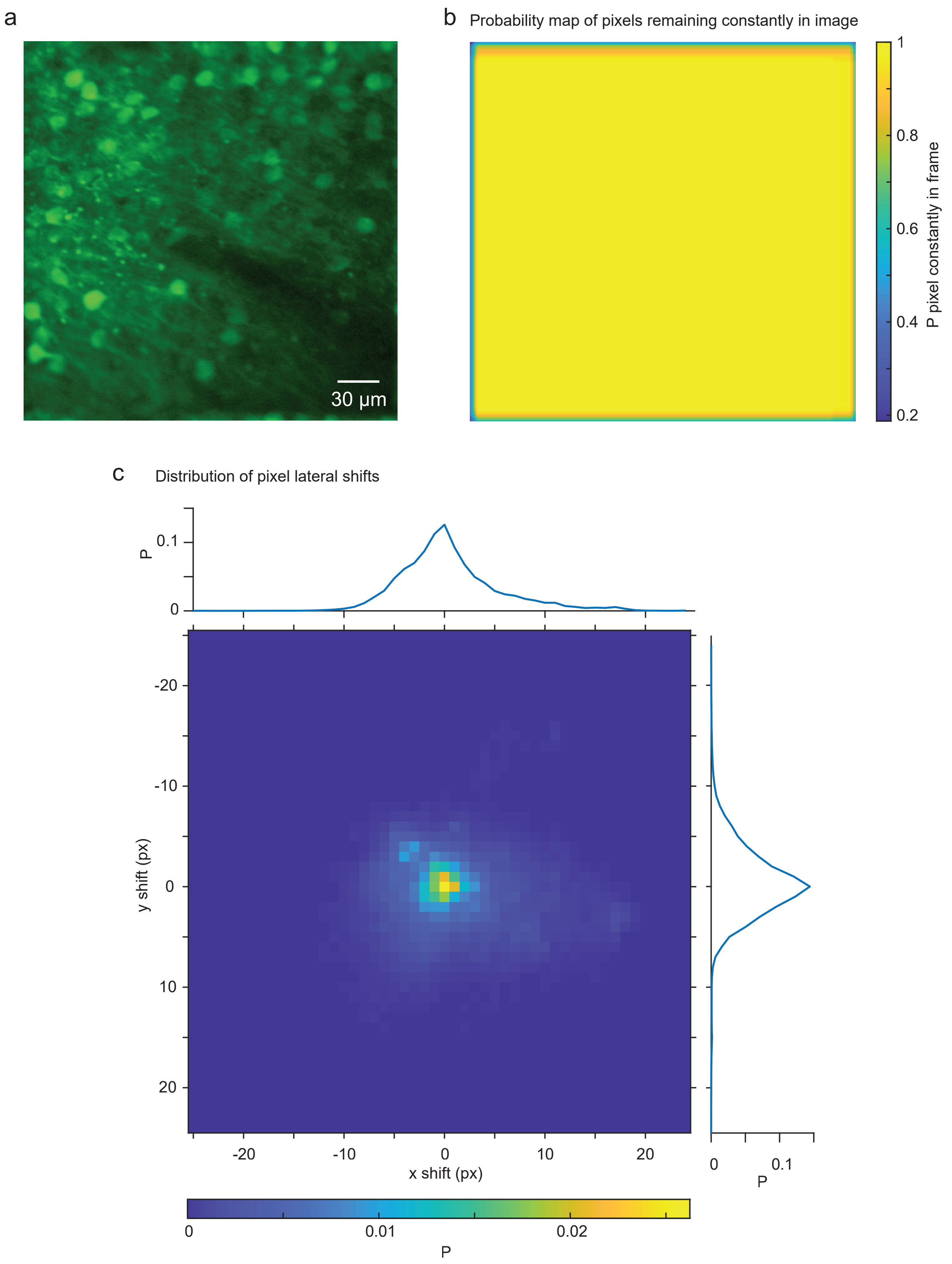

Supplementary Figure 7. Lateral stability for acquisition in freely moving mice. a, example image of a population of neurons in layer 6 for reference and comparison with panel b. Scale bar in a also applies to b. **b**, probability map showing the average probability over all data acquired in freely moving mice of a pixel being constantly in the image (not moved out of the image during motion correction). Data from 35 Ca^{2+}-imaging movies from 4 mice. **c**, distribution of lateral pixel shifts. Data as for b.

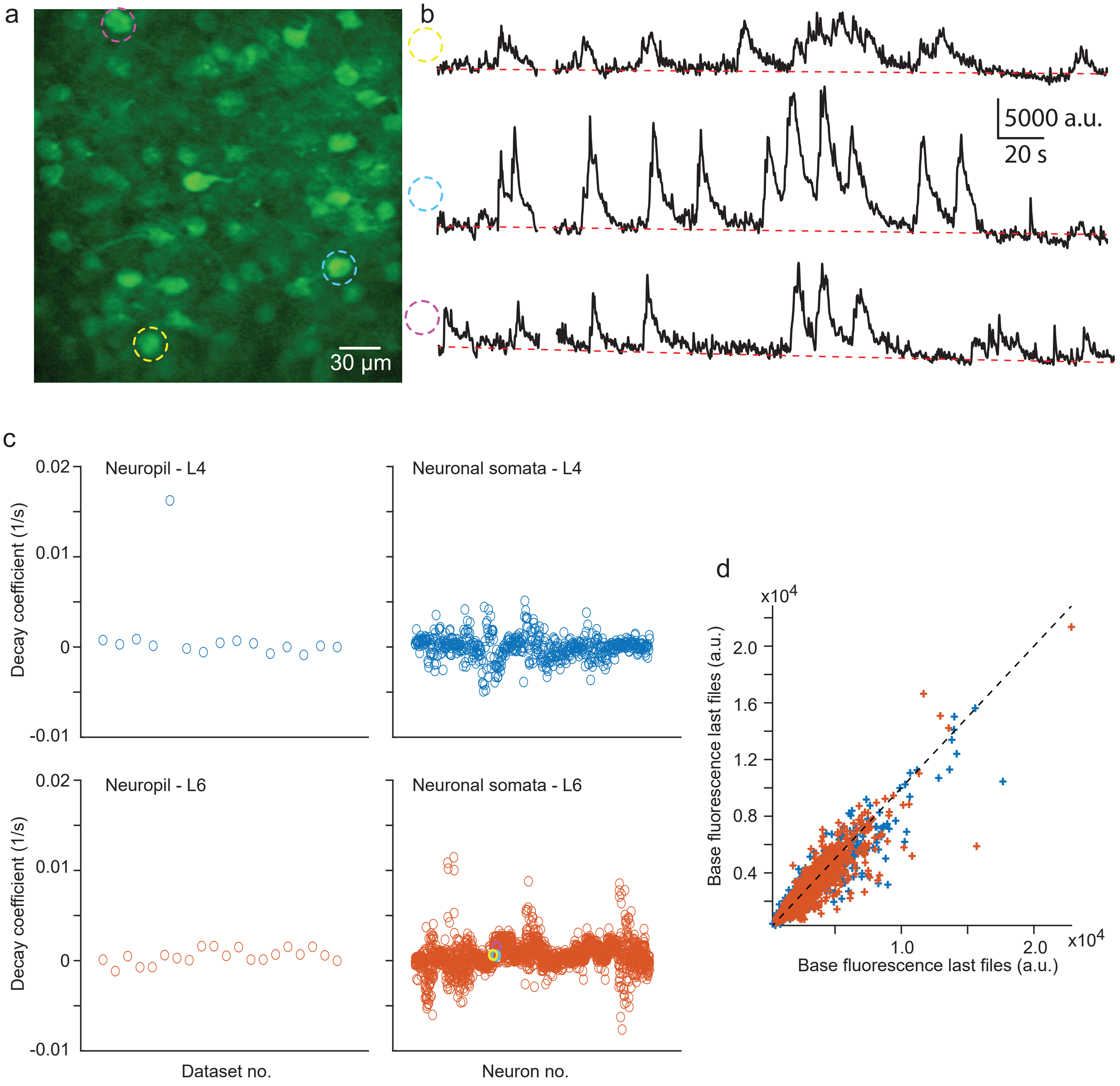

Supplementary Figure 8. Imaging does not result in photobleaching or photodamage. a, example population of neurons in layer 6. Example data traces from the neurons marked with the dashed circles shown in b. **b**, example raw data traces after de-noising from the neurons identified in a. Red dashed lines show exponential fit to baseline fluorescence (20 percentile in a 50 s sliding window). Laser power for a and b 40 mW. **c**, Exponential decay coefficients for neuropil-related fluorescence (left column) and neuronal somata (right column) for all datasets in layer 4 (upper) and layer 6 (lower). Neurons in a and b shown in corresponding color in c, right, lower. Decay coefficients for neurons denoted by yellow, blue and violet dashed circles 0.00063, 0.00057 and 0.0015 respectively. Data from 15 L4 and 20 L6 Ca^{2+}-imaging movies, and from 469 and 1295 neuronal somata in layer 4 and 6 respectively, from N=4 animals. **d**, relation between average baseline (20 percentile) fluorescence after de-noising from neuronal somata in layer 4 (blue) and layer 6 (orange) in the first (ordinate) and last (abscissa) 10% of recorded data Ca^{2+}-imaging movies.

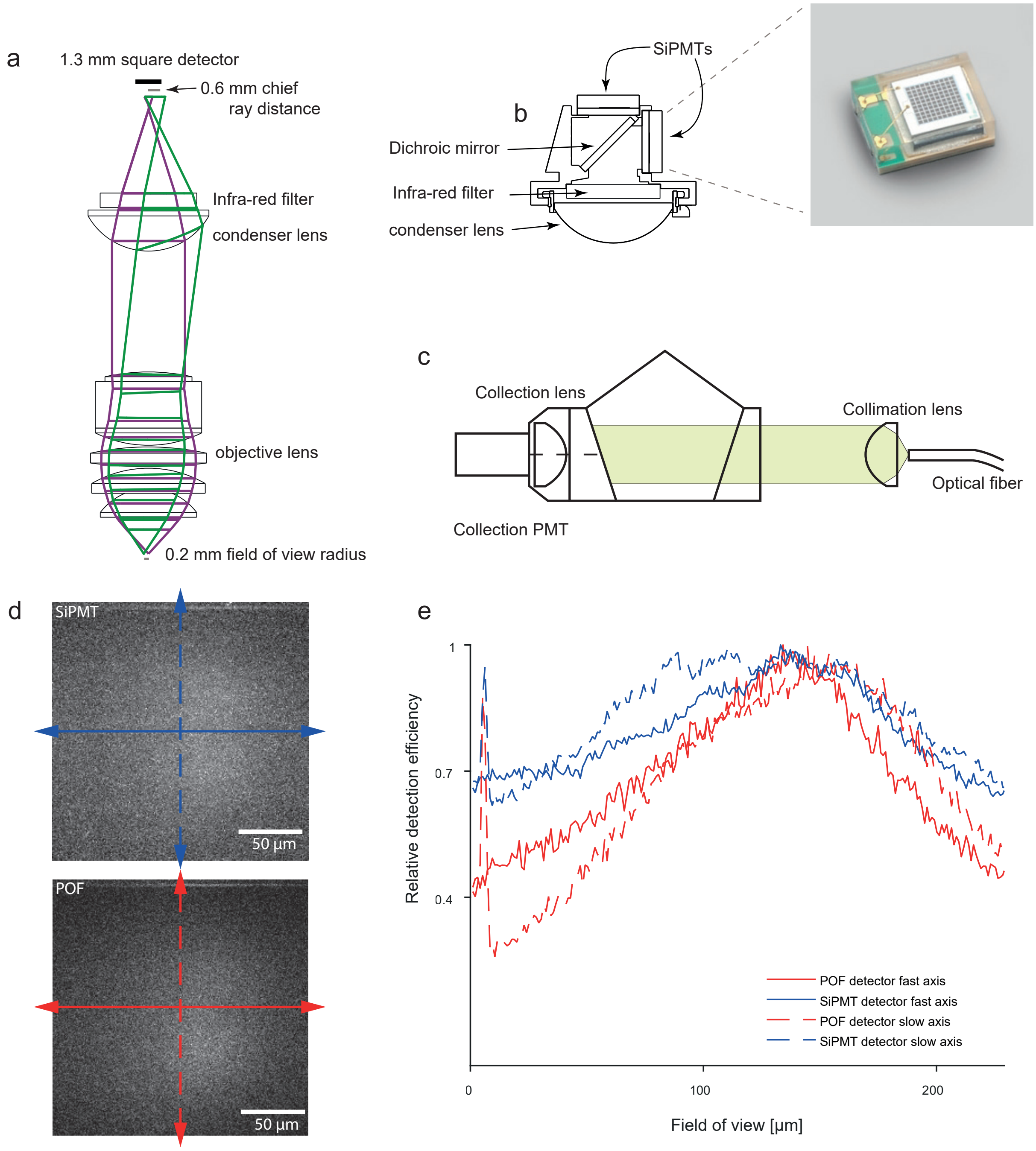

Supplementary Figure 9. Detection system utilizing SiPMTs. **a**, optical simulation of the detected fluorescence with 0.9 collection NA and 200 µm radius field-of-view. Gray lines represent one radius of field-of-view (bottom) and distance between chief rays of fluorescence detected on-axis and 200 µm off-axis at the detector plane (top). The detector surface is also shown in black, for comparison. Note that some vignetting appears in the extreme region of the field-of-view. **b**, mechanical layout of the 2-channel detector system (left) and photograph of a SiPMT detector (right). **c**, detector system layout employed for detecting fluorescence through the plastic optical fiber. **d**, average of 200 frames of imaging fluorescein solution under identical conditions with the SiPMT based detector system (top) or plastic optical fiber based detection system (bottom). Both images are on the same look-up table. The axes shown are used to calculate projection profiles in e. The bright stripe in the top region of both images is the flyback period. **e**, relative detection efficiency through the axes shown in d, for the SiPMT- (blue) and POF-based (red) detection. Each curve has been normalized to its maximum. The maximum detection efficiency for both schemes is compared in Figure 2b. Note the peak on the side of the field-of-view is due to the flyback.

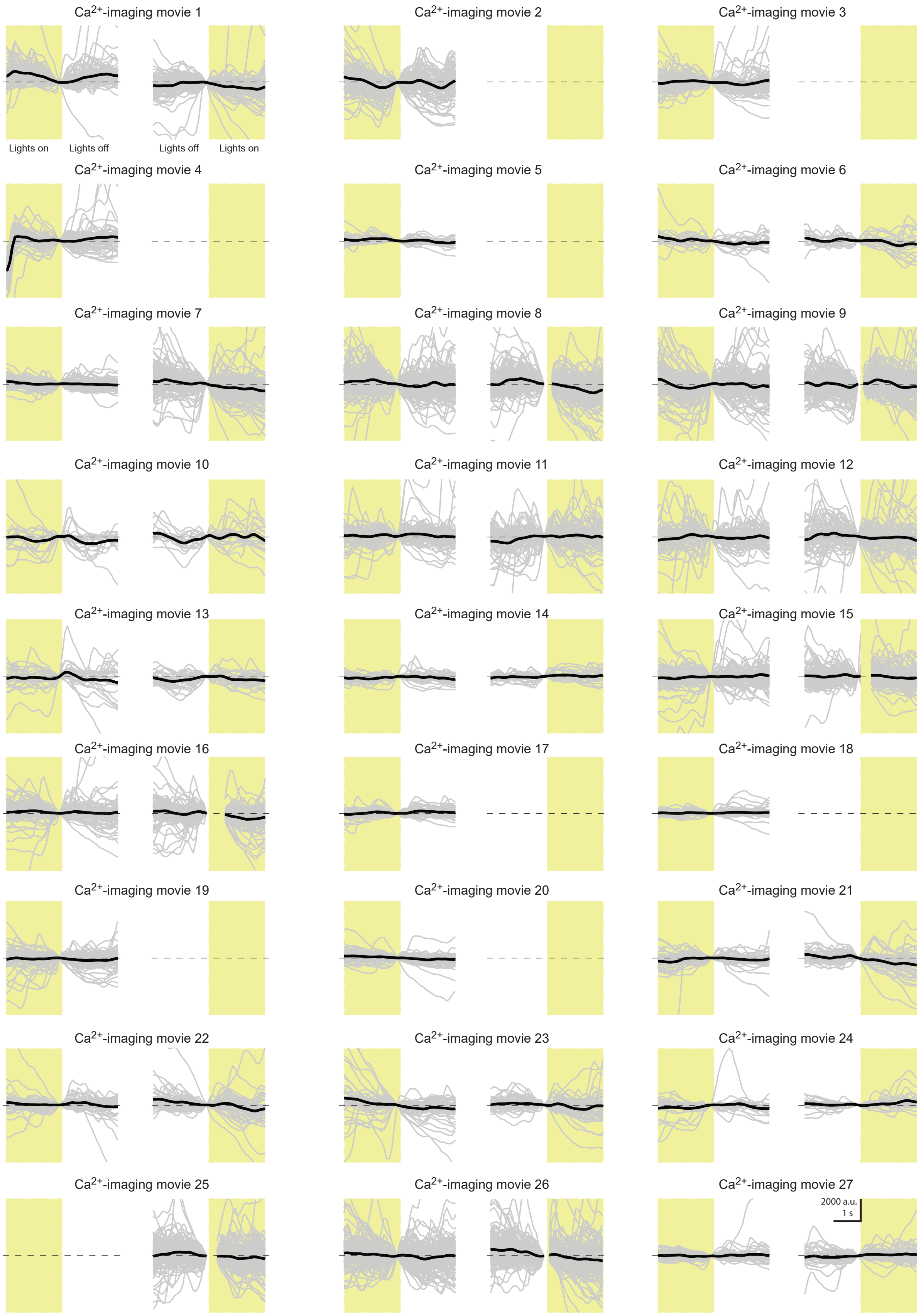

Supplementary Figure 10. Turning ON or OFF the environment light does not produce systematic offset in the fluorescence recording. Compiled de-noised jGCaMP7f fluorescence traces from all neurons in all the Ca^{2+}-imaging movies over environmental lighting transitions from on to off (left side of each panel) or off to on (right side). Traces have been normalized to the time point one frame prior to the time the environmental lights were inactivated/activated. Gray traces are traces from individual neurons, black traces represent the average. Yellow boxes represent periods with the environmental lighting on, white background represents periods with the environmental lighting off. Scale bar in the bottom right panel applies to all panels.

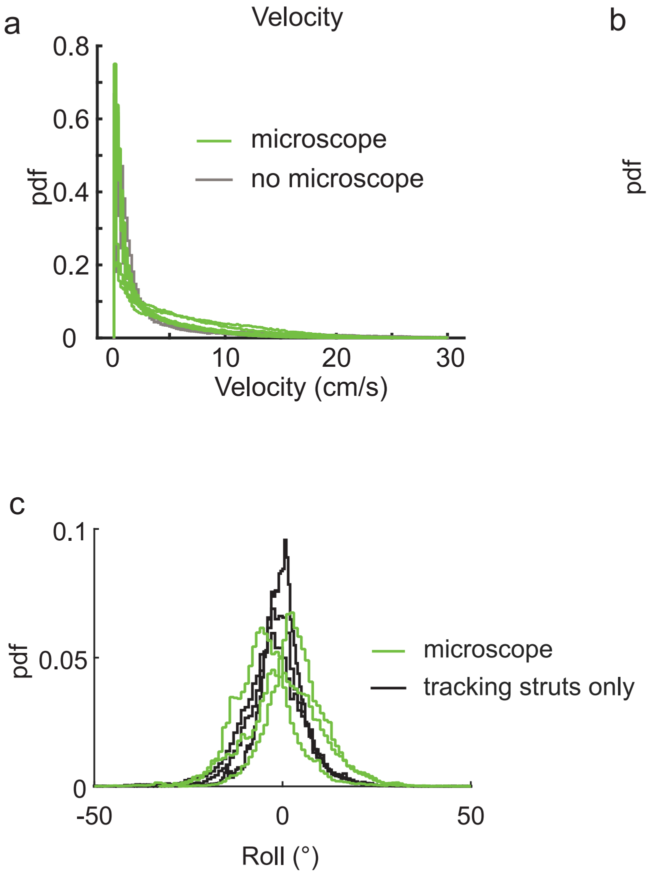

Supplementary Figure 11. Head pitch and roll is not significantly different with or without microscope. a, animal velocity distribution with (green) and without microscope (grey). Median velocity with and without the microscope was not significantly different (median ± SD, naïve mice, 10.72 ± 1.75 cm/s, N=3 animals, carrying microscope 8.43 ± 0.70 cm/s, N=8 animals, p = 0.28, Wilcoxon rank sum test). Data with microscope is from all animals imaged (N = 8 animals) and data from without microscope from separate set of naïve animals (N = 3 animals). **b**, head pitch distributions for animals with tracking struts alone (black) and with microscope (green). **c**, head roll distribution for animals with tracking struts alone (black) and with microscope (green), same data as in b. Median values for head pitch and head roll were not significantly different between the animals carrying the microscope or tracking struts alone (median ± SD, pitch, micro. -17.20 ± 5.03 °, struts. -19.41 ± 8.06 °, p=0.7; roll, micro. 0.17 ± 4.30°, struts. -1.50 ± 1.29 °, p=0.7; N=3 animals in all cases, Wilcoxon rank sum test in all cases).

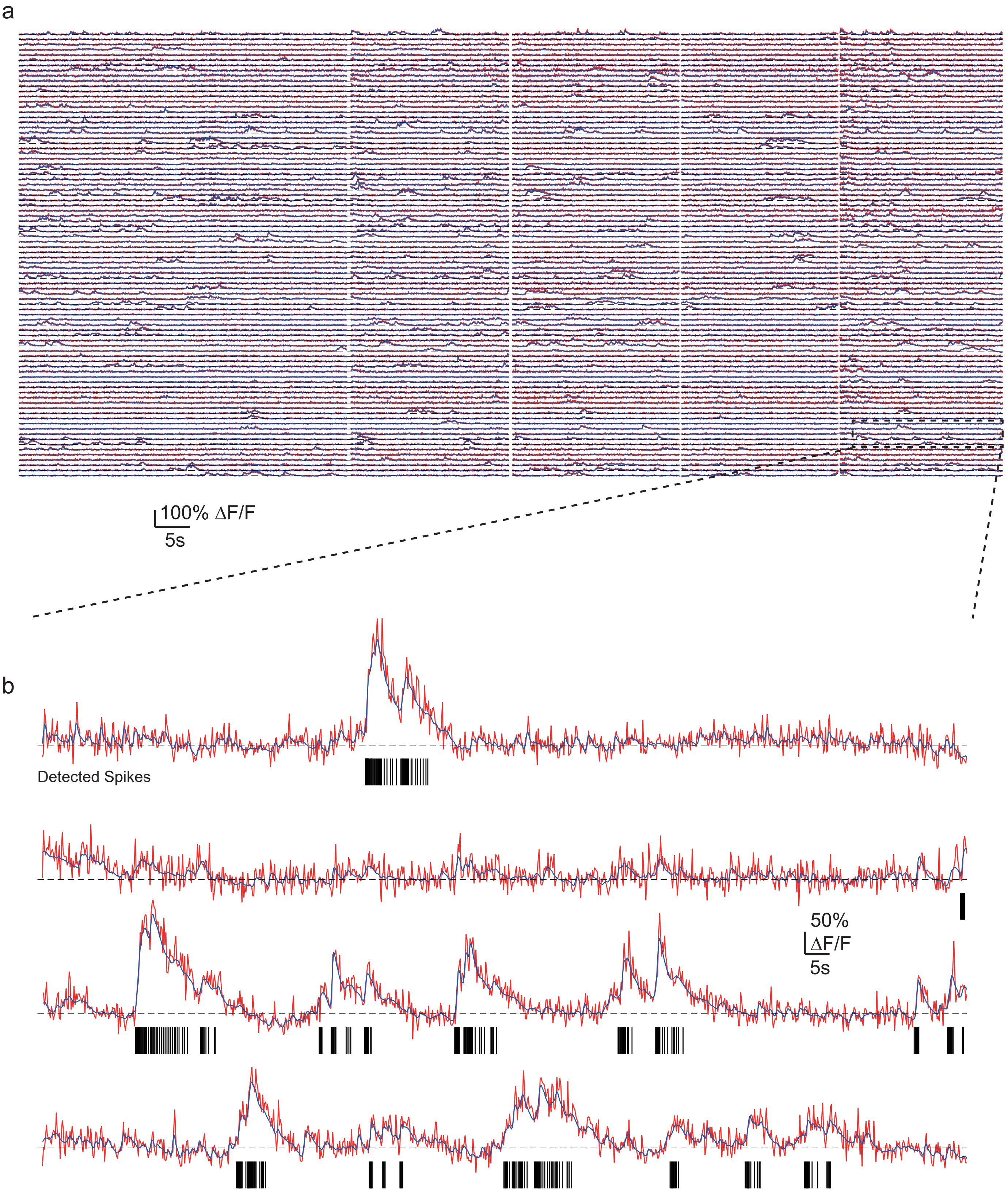

Supplementary Figure 12. Example performance of de-noising and spike finding algorithms on acquired data. a, neuronal activity from all neurons during one recording session in cortical layer 6, showing raw (red) and de-noised (blue) traces from each neuron, with estimated baseline for each neuron (black dashed). **b**, enlarged portion of indicated region in a. Vertical lines represent inferred spike times from ML spike. Color scheme as in a.

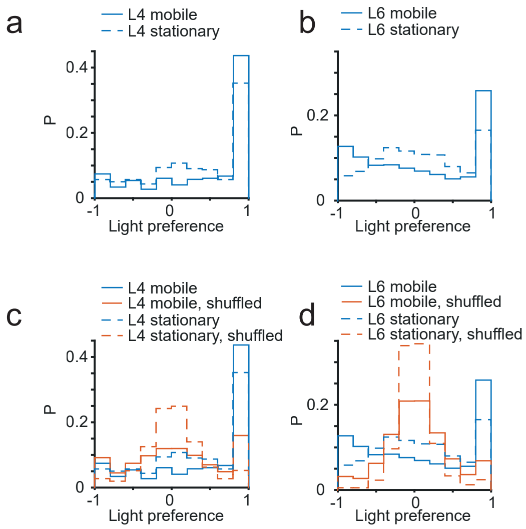

Supplementary Figure 13. Light preference of neurons when the animal is mobile or stationary. **a**, distribution of light preference indices for L4 neurons from all datasets (N=298 neurons from 9 datasets from 3 animals) with animal mobile (solid) or stationary (dashed). **b**, as for a, but for all neurons from all datasets in L6 (N=880 neurons from 9 datasets from 4 animals). **c**, same as a, with superimposed shuffled data. **d**, same as b, with superimposed shuffled data.

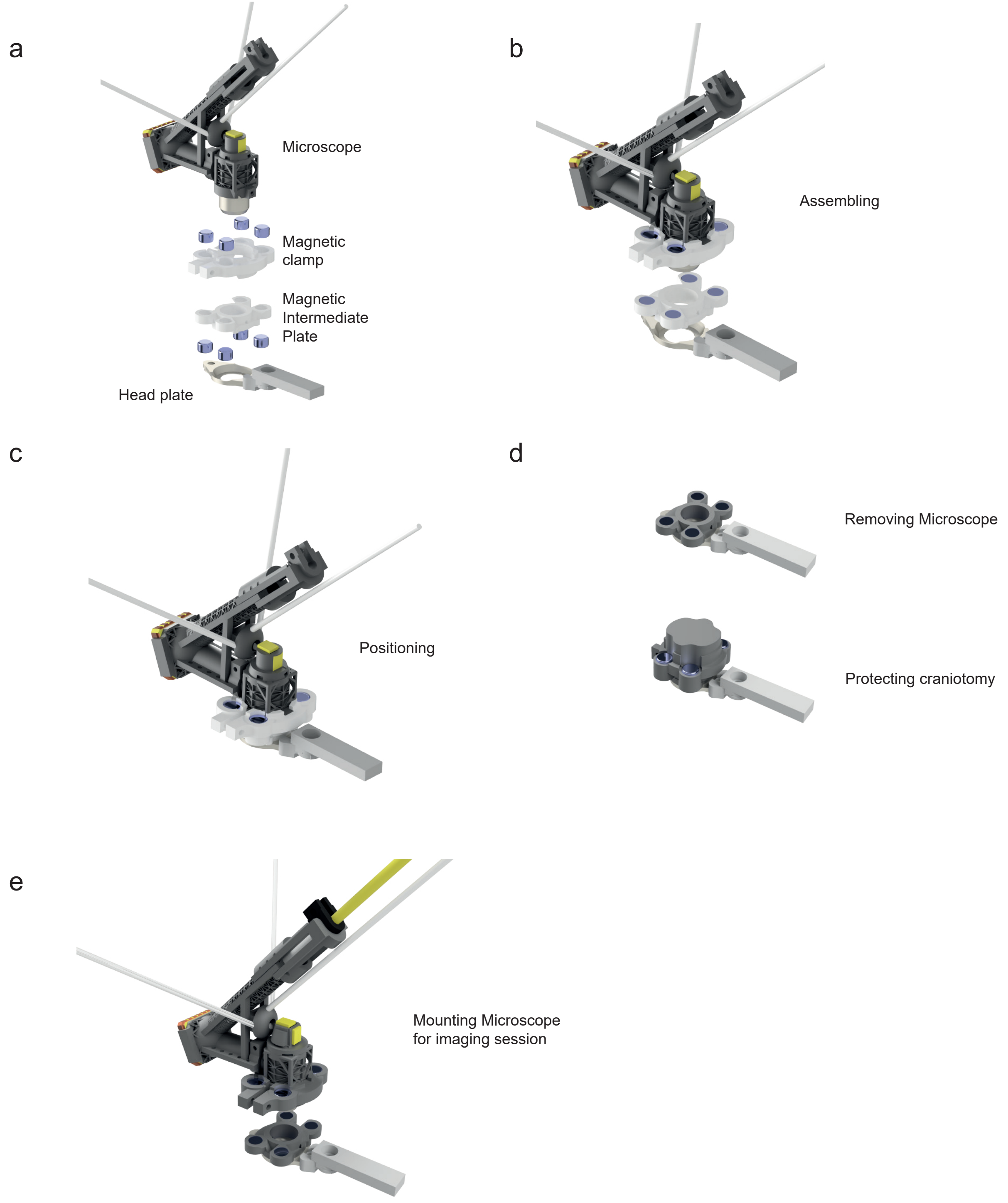

Supplementary Figure 14. Headplate, intermediate attachment plate and procedure for mounting the microscope on the headplate. a, exploded view of the mounting parts. **b**, microscope mounted in the magnetic clamp prior to attachment of the magnetic intermediate plate. **c**, microscope with magnetic clamp and magnetic intermediate plate attached as mounted into the 3D positioning system. The positioning system allows navigation around the cranial window and positioning over a clear field-of-view of neurons. **d**, after positioning, the intermediate plate is fixed to the head plate, the microscope removed and the intermediate plate covered with a protective cover. **e**, microscope and magnetic clamp assembled for mounting the microscope for an imaging session. See also Supplementary video 6.

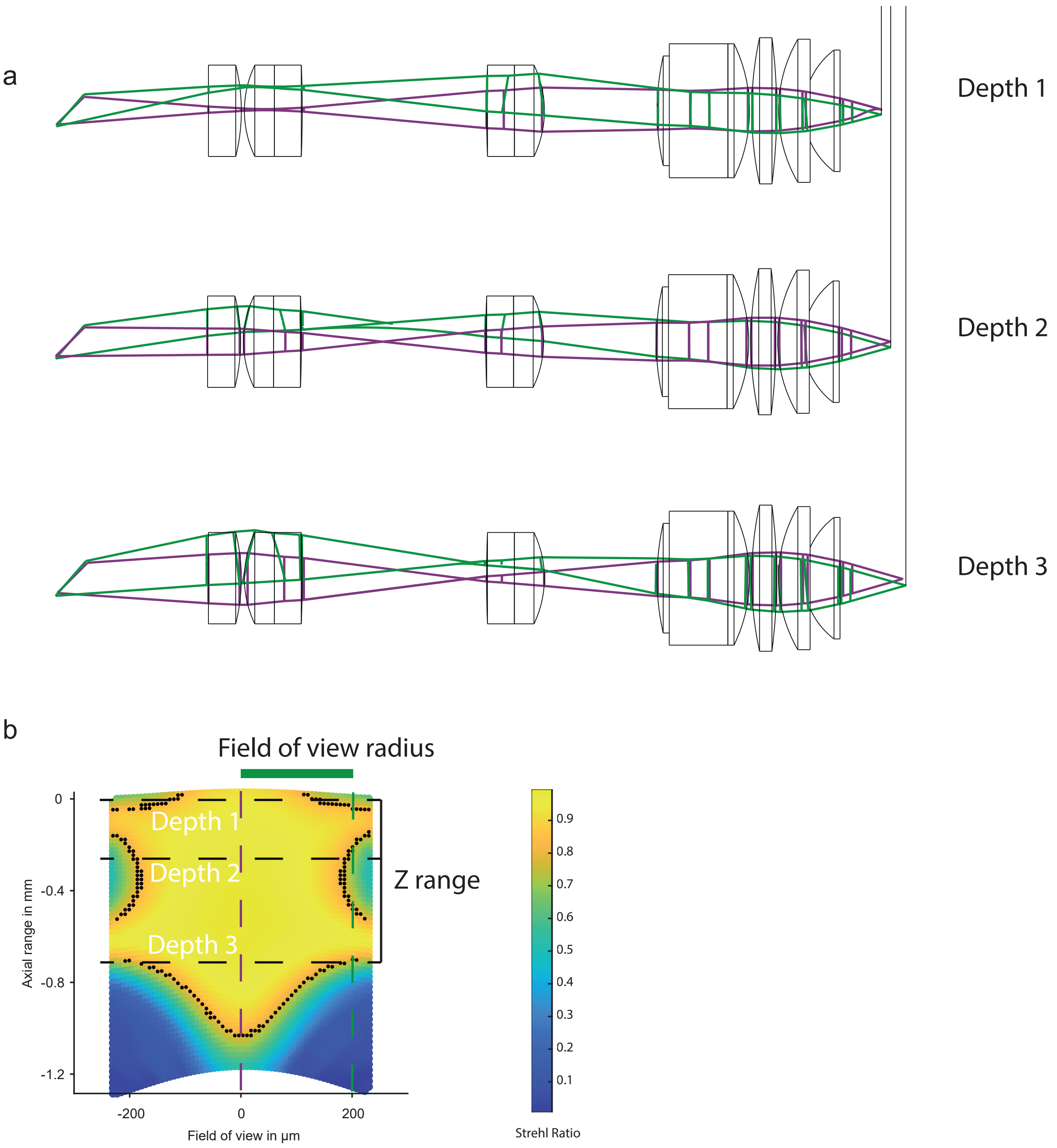

Supplementary Figure 15. Initial step in the optical design: scanning module under multiple defocus conditions. a, optical layouts illustrating three configurations with different defocus applied to the input beam. The first surface modeled (left of image) is the MEMS scanner mirror. On-axis and maximal mirror deflection (extreme side of imaged area) beams are shown in violet and green respectively. This system corresponds to the original optimization conditions. **b**, simulated optical performance measured by polychromatic Strehl Ratio (SR, color-coded) over the field-of-view and the z-range achievable by changing the fiber-collimation lens distance. Black dots delimit the diffraction limited imaging area (SR > 0.8). The on-axis and maximum deflection for field-of-view positions are indicated by the violet and green dashed lines, and the three depths from a by the black dashed lines.

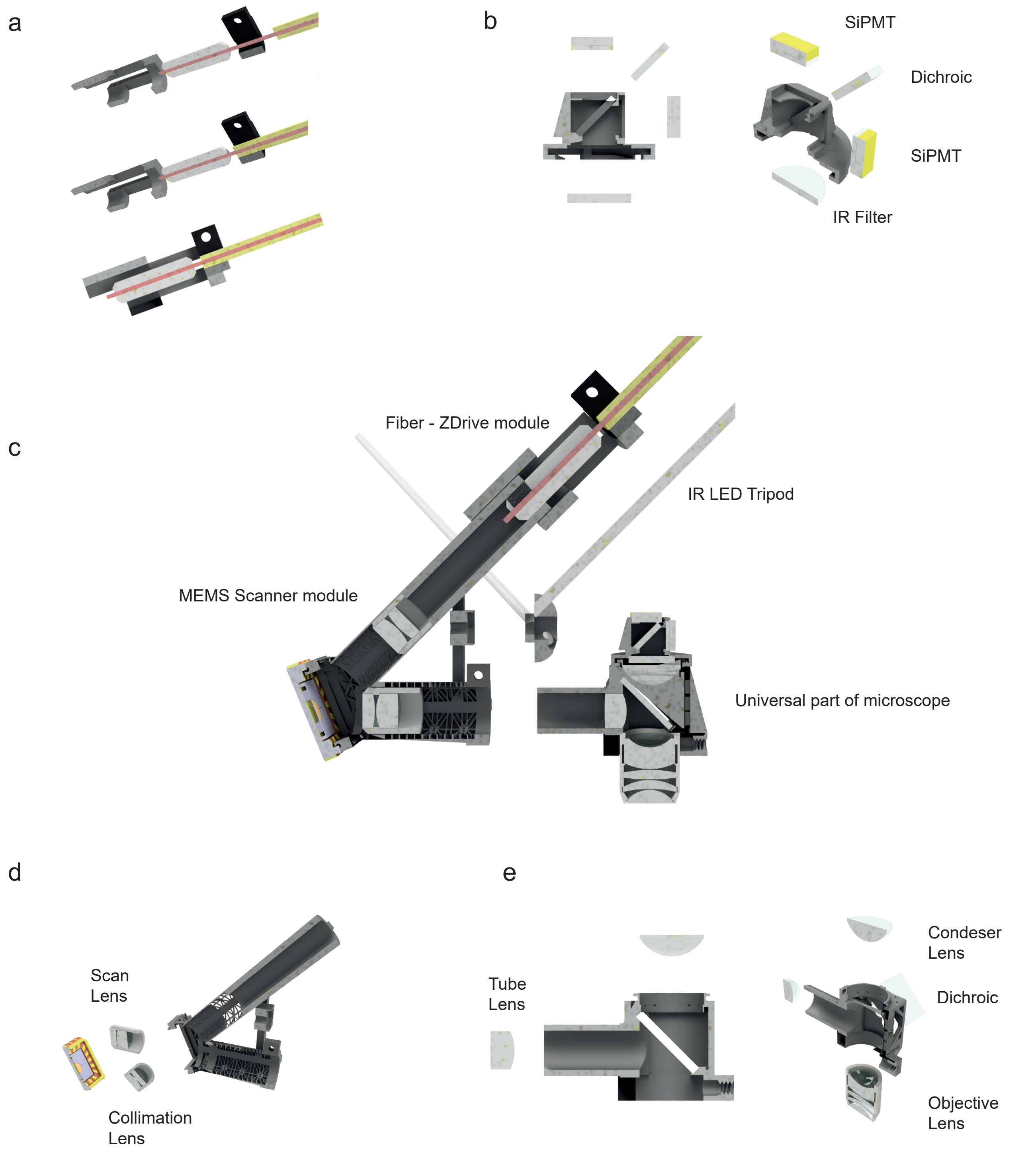

Supplementary Figure 16. Building the microscope: profile and oblique views on sections of all microscope components. a, fiber z-drive module mounting: first the ferrule is inserted, then the jacket is pressure fitted in the attachment part, finally the part is attached together. **b**, detector system. First the dichroic mirror is inserted through the top surface. Optional fluorescence filters are glued on top of the detectors, and then the detectors are glued inside their compartments. Finally the infra-red (IR) filter is inserted into the bottom aperture. **c**, the final microscope assembly step is to join together the MEMS scanner module, with LED tripod attached to it, and the universal microscope part (identical for all of the microscope versions). Most of the parts and optics hold sufficiently well with pressure fit. In rare occasions it is necessary to add some glue, as the tolerances of 3D printing and different parts are not good enough to ensure a stable press fit in all cases. **d**, MEMS scanner module. First the collimation lens assembly is inserted, then the scan lens assembly, then the MEMS scanner. In our experience fitting the MEMS scanner in its socket is sufficient and no further alignment is necessary. **e**, universal microscope part. The dichroic mirror is first inserted from the side. Then the tube lens is positioned through its aperture. The condenser lens is added from top. The objective lens can be mounted and dismounted anytime and is usually added just before an imaging session. See also Supplementary video 7.

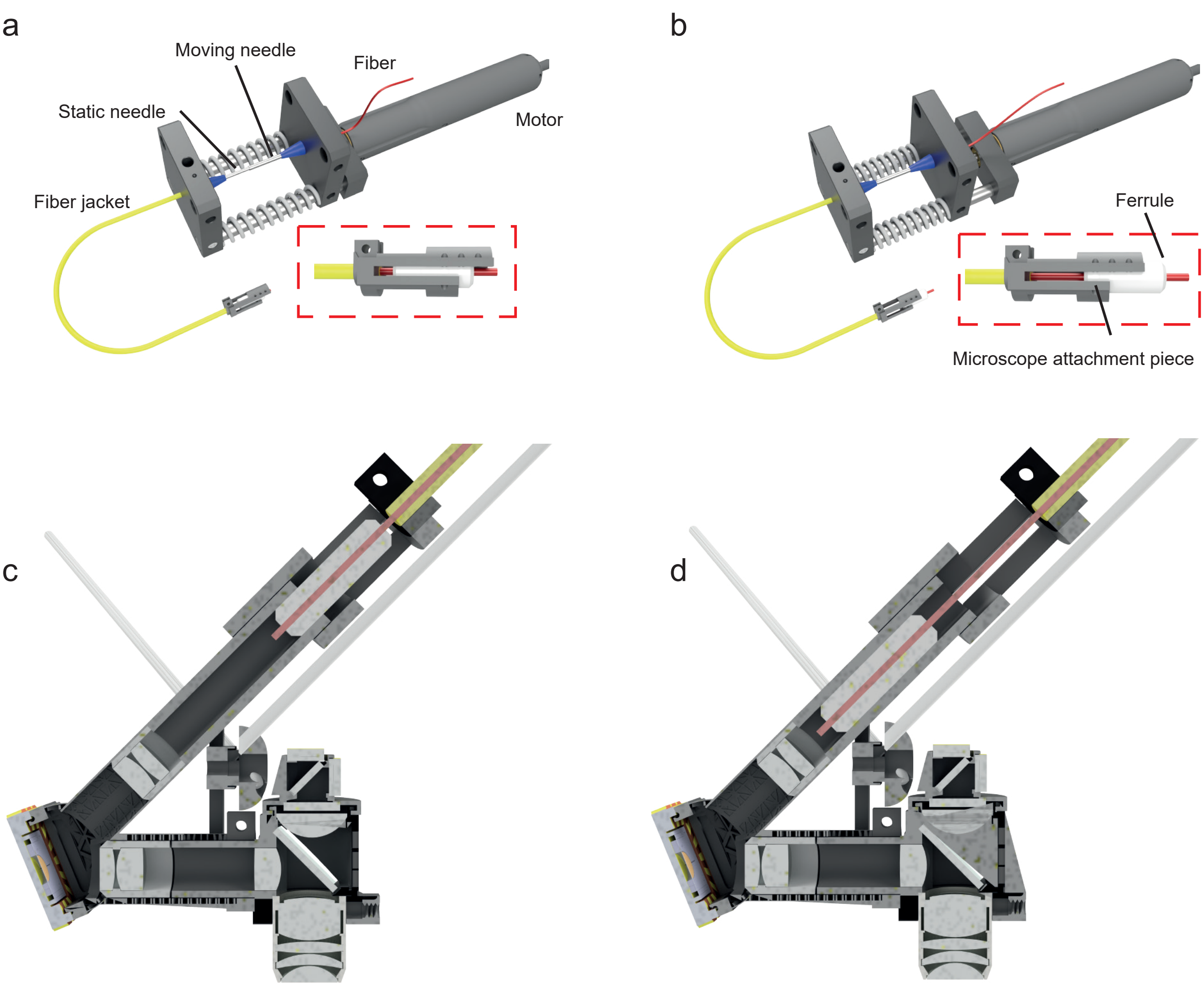

Supplementary Figure 17. Implementation of the motorized mechanical fiber tip shift actuated from the laser table side of the fiber. a, motorized optical table setup for actuating the fiber tip, with the fiber tip, glued inside a ceramic ferrule, in its most retracted position. The inlay shows an enlarged view of the ferrule and microscope attachment piece. **b**, as in a, but with fiber tip in its most extended position. **c**, cross-section of the microscope with the fiber passively sliding in a cylindrical sleeve, show in its most retracted position. **d**, as in c, but with the fiber tip in its most extended position. See also Supplementary video 8.

Supplementary Text

Surgical implants and mounting devices

The equipment used to mount the microscope on the head of the animal for freely moving experiments consisted of a surgically implanted headplate, an intermediate attachment plate and a microscope adapter plate (Supplementary Fig. 14, Supplementary video 6), all of which were custom designed and fabricated.

The implanted headplate (Supplementary Fig. 14) was 3D-printed using Temporary CB (Formlabs, MA, USA) and had a weight of ~100 mg. A handle, 16 mm in length, was 3D-printed using black resin V4 (Formlabs, MA, USA) and attached to the headplate using UV-curing dental adhesive (Charisma Flow, Kulzer GmbH, Hanau, Germany). The base of the headplate had a 5.3 mm diameter aperture below which the craniotomy for imaging was opened (details below). A hole with an independently 3D-printed tapped insert at the front of the headplate was used for attaching a removable crossbar for stable fixation during surgical procedures or allowed securing a protective cover when the mice should be placed in their cage.

The intermediate attachment plate (Supplementary Fig. 14), allowed alignment of the microscope over the craniotomy and the region of interest before being rigidly attached to the headplate, again using UV-curing adhesive (Charisma Flow, Kulzer GmbH, Hanau, Germany). The microscope magnetic clamp (Supplementary Fig. 14) had a central aperture that fitted tightly around the microscope, secured by a screw.

Both the intermediate attachment plate and the microscope magnetic clamp were equipped with 4 neodymium magnets each (⌀3 mm, height 2 mm, Webcraft GmbH, Gottmadingen, Germany), providing up to 24 N holding force. Additional opposed guide fins provided stable orientation. This design allowed fast and reliable placement and detachment of the microscope during experiments. The weight of the intermediate attachment plate, including magnets was 575 mg. The weight of the microscope magnetic clamp, including magnets was 630 mg.

All components were silanized (Rotatec bonding system, 3M ESPE, Neuss, Germany) prior to each use to improve surface adhesion.

Surgical procedures for fluorescent labelling of neurons with jGCaMP7f

Prior to surgery, all instruments, including the glass injection capillary, were sterilized by either autoclaving or heat sterilization. Animals were anesthetized with an intraperitoneal injection of a three-component anesthetic cocktail (3K) consisting of fentanyl (50 µg/kg, Hameln pharma plus GmbH, Hameln, Germany), midazolam (5 mg/kg, Hameln pharma plus GmbH, Hameln, Germany) and medetomidine (0.5 mg/kg, Zoetis, NJ, USA). Body temperature was maintained at 37–37.5 °C with a heating pad and heater controller (FHC, ME, USA). Animal status and depth of anesthesia was monitored approx. every 15–30 min, and anesthesia maintained throughout with supplementary doses of 30–80% of the above anesthetic combination given as necessary in order to maintain absence of withdrawal and corneal reflexes. The animals were then placed in a stereotaxic apparatus, the hair on the scalp removed and the skin cleaned with 70% ethanol. The right parietal bone was exposed and a burrhole drilled (approx. 500 µm diam.) at 3.0 mm posterior and 2.6 mm lateral relative to bregma. A small slit was made in the dura underlying the burrhole, and a glass injection capillary with a beveled tip containing a

high-titer solution of AAV1/2 coding for Cre-dependent jGCaMP7f (AAV1/2.hSyn.FLEX.jGCAMP7f, Addgene, MA, USA) was advanced posteriorly into the cortex approx. 1650 µm at an angle of 25° relative to horizontal along a trajectory parallel with the saggital suture. An injection of approx. 180 nL of the virus solution was made with a nanoliter injection device (Nanoject II, Drummond Scientific Company, PA, USA). After a delay of 5 min, the tip of the capillary was withdrawn approx. 240 µm, and an injection of approx. 115 nL made. After another delay of 5 min, the tip of the capillary was again retracted approx. 240 µm, and another injection of approx. 115nL made. After a final waiting period of 5 min, the glass capillary was slowly withdrawn, the craniotomy covered with medical silicone (KwikSil, WPI, FL, USA) and the skin sutured closed using 5/0 vicryl sutures (Ethicon, NJ, USA). Animals were then administered buprenorphine (30 µg/kg, Bayer, Leverkusen, Germany) and carprofen (5 mg/kg, Zoetis, NJ, USA) for post-operative analgesia, and then a cocktail of antagonists to the anesthetic drugs (anti-3K) consisting of naloxone (11.2 mg/kg, Ratiopharm, Ulm, Germany), flumazenil (0.5 mg/kg, Hikma, Amman Jordan) and atipamezole (0.75 mg/kg, Orion Pharma, Hamburg, Germany).

Linear track and animal position and orientation tracking

The linear track was 96.0×9.7 cm^2, surrounded by a rim 2 cm in height and raised approx. 110 cm off the floor. The animal was free to explore and move around the track during experiments. Animal position and head orientation were tracked as described in [1]. In brief, 3 struts were mounted onto the body of the miniature microscope, with 3 infrared (IR) LEDs (940 nm, SFH 4053, Osram) mounted on each. Images of the IR-LEDs were acquired at 200 Hz by 4 calibrated and synchronized digital cameras (acA1300-200um, Basler AG, Ahrensburg, Germany) mounted above the track. The cameras were equipped with 8.5 mm/F1.5 objective lenses (Pentax, Ricoh Imaging, Tokyo, Japan) to facilitate coverage of the track and IR bandpass filters (LP 780-40.5, Midwest optical systems, IL, USA) to facilitate automated tracking of the LEDs in the images. The exposure active signals from the overhead tracking cameras as well as the frame synchronization signal from the miniature microscope were fed into an analog-digital converter (Power 1401, Cambridge electronic design, Cambridge, UK) and recorded with Spike-2 software (version 6.18, Cambridge electronic design, Cambridge, UK) for synchronization of behavioral tracking and multiphoton imaging data. Determination of animal position and head orientation were performed using the method described in detail in[1], initialized by a pre-detection step using multitrackpy (https://github.com/bbo-lab/multitrackpy). Mounted directly next to each of the above cameras was a second camera (acA1300-200um, Basler AG, Ahrensburg, Germany), equipped with an 8.5mm/F1.3 lens (Edmund optics, New Jersey, USA) for simultaneously recording the animals position on the track at the visible light level. All cameras were calibrated using the calibration procedure described in[2].

Histology

At the termination of the imaging experiments animals were deeply anesthetized with ketamine (100 µg/kg) and medetomidine (200 µg/kg), and perfused transcardially with 0.1 M phosphate buffer (PB) followed by 4% formaldehyde solution (Roti-Histofix, Carl Roth, Karlsruhe, Germany). The brain was then removed, post-fixed at 4°C overnight in the same formaldehyde solution and then transferred to 0.1 M PB. Sections of 100 µm thickness were then cut on a vibrating microtome (Leica VT1000S, Wetzlar, Germany) and mounted in fluoroshield (Sigma, MO, USA). Images were acquired on an inverted microscope (Nikon Ts2R-FL, Tokyo, Japan).

Comparison of SiPM and POF detection system signal to noise

The comparison between the SiPM-based detector system and the plastic optical fiber and PMT detector system (as described in Klioutchnikov et al. 2020[3]) were performed in a dark environment, with both detection systems using identical amplifiers and acquisition systems, and identical image gating settings for the acquisition gating system described in the current manuscript. The sample was a fluorescein solution to provide a uniform sample and prevent bleaching from influencing the comparison. Laser power was selected such that the measured signal approximately matched the average signal of neurons in typical experiments. For analysis of the datasets, five regions of interest with increasing sizes (1, 4, 16, 64 and 259 pixels) were defined and placed in the highest signal region of the images from both detector types. Signal-to-noise ratio (SNR) was calculated for fluorescence signal integrated over these regions as signal mean divided by signal standard deviation. For acquisitions using the SiPMs, image pixel value histograms show defined peaks for 1, 2, 3, 4 and 5 detected photons, allowing quantification of detected photons. For the data presented in figure 3c, there was an average of 0.334 ± 0.777 (mean ± SD) photons per pixel per frame. In the same dataset, regions of interest containing 1, 4, 16, 64 and 259 pixels had 0.39, 1.65, 5.7, 22 and 86 detected photons per frame respectively.

Volumetric imaging

The mechanical (fiber-translation-based) focusing mechanism has been designed to provide on-demand access to all layers in the mouse cortex while maintaining stable imaging, with a maximum velocity of movement of approx. 100 µm/s, making it not suited to volumetric imaging. The electrically tunable lens (ETL) had a settling time of around 2 ms, making it suitable for volumetric imaging, though the maximum z-range is reduced compared with the mechanical focusing mechanism and the microscope weight increased, as described in the Results subsection "*Light weight microscope with remote focusing to image all cortical layers*".

Data analysis

Alignment of single frames for motion correction

Motion correction was performed using the image registration component of suite2p[4] (parameters: align_by_chan=1, nimg_init=200, batch_size=5000, smooth_sigma=1.15, maxregshift=0.1, nonridid=0), involving a displacement estimation using phase-correlation and subsequent rigid frame shift. In addition to the corrected movie, the algorithm yields the per-frame-displacements in pixels for horizontal and vertical direction respectively, which have been used for the quantification of brain movement.

Extraction of fluorescence signal

The fluorescence F_t in the tth frame for a given neuron was extracted by calculating the per-frame-average of pixel values in a region of interest (ROI). ROIs where manually drawn around the somata of cells in focus and linked over a continuous recording session of multiple files.

Calculation of $\Delta F/F_0$

The baseline fluorescence $F_{0,t}$ was defined as the mean of the lowest 20% of fluorescence values within a window of maximally 107.63s around the tth frame (if not truncated by beginning or end of the file) after applying a Gaussian filter with a standard deviation of 282ms, and

$$\Delta F_t/F_{0,t} = (F_t - F_{0,t})/F_{0,t}.$$

Trace de-noising

For de-noising the raw data we used DeepCAD-RT (Git version fe68f28 from Aug 26, 2021, https://github.com/cabooster/DeepCAD-RT) with the default "ModelForPytorch" model.

Analysis of photo-bleaching and photo-damage

For quantification of photo-bleaching, we considered each de-noised recoding file individually and created a background mask using a sliding 20% percentile window with a width of 50s. We then fitted an exponential function of the form a*exp(-b*x) to the identified background using MATLAB's fit function, with parameter b being taken as the stated decay coefficient. This procedure was performed for each neuronal trace in each data file, as well as a 'neuropil' trace for each file consisting of the average value from all pixels not included in neuronal regions of interest.

For quantification of photo damage, we first extracted the 20th percentile of the first and last 10% of each de-noised recording file, and then calculated the relative difference (first-last/(first+last)) of these two values.

Spike detection

Spike detection was performed using MLspike[5] (available at https://github.com/MLspike/spikes,%20commit%2032fb84e) on dF/F_0, using parameters

F0 = [-0.5 1.3], a = 0.034, pnonlin = [0.85 -0.006], drift.parameter = 0.015, finetune.sigma = 0.08, algo.nc = 50, algo.nb = 50. Since no parameters for jGCaMP7s are available, these parameters were based on the ones for GCaMP6f and manually inspected for consistency with the fluorescence signal. While consistency of active and inactive period is well maintained between transients and inferred spikes, absolute numbers of spiking rates are to be interpreted with care.

Shuffling of spikes

To gauge the significance of the observed light-dark responses, we artificially shuffled the recorded frames to create a control group of random firing. To maintain the original structure in the firing, we shuffled as follows: First, we created a list of inter-spike intervals (ISI), consisting of the intervals between spike times, and the sum of the beginning and end intervals between recording start and end and the first and last inferred spike, respectively. Subsequently, we separated this list into a list of short (<= 3*dt) ISIs and list of the remaining longer ISIs. We then created a list of spike train lengths, that is, numbers of spikes that are only separated by short ISIs. The list of long ISIs was summed and the total time was randomly redistributed to the same amount of ISIs.

Finally, we constructed a new, randomized spiking pattern by randomly sampling (without replacement) from the lists of train lengths, short ISIs (for the ISIs within a spike train) and long ISIs (for the ISIs

between spike trains). The initial interval before the first spike train was drawn from the list of long ISIs and subsequently multiplied by a random number between 0 and 1.

We used this method 100 times on the original data to create 100*n randomly firing neuron.

Image plane separation in multiplane imaging dataset

In the multiplane imaging dataset presented in Fig. 2e-g and Supplementary Fig. 5, the imaging plane was manually shifted until the neurons in the field of view were different. Based on the diameter of the neurons in the overview images and the histological section in figure 2 we estimate that the approximate diameter of the neurons in the field of view is around 15 µm. Shifting the focus position until the field of view was different suggests that the image plane had been shifted by approximately half to one neuron diameter, equating in these experiments to between 7 and 15 µm.

Naïve mouse behavioral position tracking

For analysis of animal position without tracking LEDs, we first subtracted the background, calculated as the median of the first 100 frames acquired before the animal was put on the track. We then inverted the 8 bit image, binarized it with a threshold of 20 and morphologically eroded the resulting mask by 30 pix. to remove residual noise. We then defined the animal's 2d position on the track as the center of mass of the remaining mask. The velocity was then derived by first order numerical differentiation.

Analysis of microscope and tracking-struts-only headmount behavioral datasets

To establish a coordinate system comparable between different animals and the two different position-tracking systems (that on the microscope and the tracking-strut-only headmount) we first determined the 3D coordinates of the tracking LEDs, left and right eye and tip of the nose using the close-up images of the animals acquired at the end of the behavioral sessions and associated camera calibrations. The position of each of the tracking LEDs as well as the position of the nasal corner of each eye and the position of the tip of the nose between the two nostrils was marked in the images and 3D coordinates calculated using the method described previously[1]. From the eyes and nose coordinates, we derived an anatomical forward-left-up coordinate system by calculating a pre-forward vector using the direction between mean of eyes and nose and a pre-up vector as a vector orthogonal to the pre-forward vector and the vector between the eyes. Next, the left vector was defined as orthogonal to pre-forward and pre-up. Finally, the system was rotated by 40° around the left vector such that forward vector was elevated into the bregma-lambda sagittal plane, based on the description and measurements described previously[2]. To eliminate potential confounds resulting from the two different cable bundles for the microscope and tracking-strut-only headmount applying different forces the animal's head as it's position approached the available length of the cable, we restricted the analysis of head pitch and roll to data where the animal was >0.5 cm from the edges of the track. For each dataset in the two experimental groups the median pitch, roll, velocity and total path length travelled were calculated, the compared between the two groups using a Wilcoxon rank sum test.

References

1 Wallace, D. J. *et al.* Rats maintain an overhead binocular field at the expense of constant fusion. *Nature* **498**, 65-69, doi:10.1038/nature12153 (2013).

2 Holmgren, C. D. *et al.* Visual pursuit behavior in mice maintains the pursued prey on the retinal region with least optic flow. *eLife* **10**, e70838, doi:10.7554/eLife.70838 (2021).

3 Klioutchnikov, A. *et al.* Three-photon head-mounted microscope for imaging deep cortical layers in freely moving rats. *Nat Methods* **17**, 509-513, doi:10.1038/s41592-020-0817-9 (2020).

4 Pachitariu, M. *et al.* Suite2p: beyond 10,000 neurons with standard two-photon microscopy. *bioRxiv*, 061507, doi:10.1101/061507 (2017).

5 Deneux, T. *et al.* Accurate spike estimation from noisy calcium signals for ultrafast three-dimensional imaging of large neuronal populations in vivo. *Nat Commun* **7**, doi:ARTN 1219010.1038/ncomms12190 (2016).
