# Supplementary material for: A three-photon head-mounted microscope for imaging all layers of visual cortex in freely moving mice
Source: Nat Methods. 2022 Nov 28;20(4):610–6. doi: 10.1038/s41592-022-01688-9 (PMC10089923; doi:10.1038/s41592-022-01688-9)
Supplement: Supplementary file 2 — Reporting Summary [file 41592_2022_1688_MOESM2_ESM.pdf]

Corresponding author(s): J. N. D. Kerr

Last updated by author(s): Oct 2, 2022

Reporting Summary

Nature Portfolio wishes to improve the reproducibility of the work that we publish. This form provides structure for consistency and transparency in reporting. For further information on Nature Portfolio policies, see our Editorial Policies and the Editorial Policy Checklist.

Statistics

For all statistical analyses, confirm that the following items are present in the figure legend, table legend, main text, or Methods section.

n/a	Confirmed	
☐	☒	The exact sample size (*n*) for each experimental group/condition, given as a discrete number and unit of measurement
☐	☒	A statement on whether measurements were taken from distinct samples or whether the same sample was measured repeatedly
☐	☒	The statistical test(s) used AND whether they are one- or two-sided<br>*Only common tests should be described solely by name; describe more complex techniques in the Methods section.*
☒	☐	A description of all covariates tested
☒	☐	A description of any assumptions or corrections, such as tests of normality and adjustment for multiple comparisons
☐	☒	A full description of the statistical parameters including central tendency (e.g. means) or other basic estimates (e.g. regression coefficient) AND variation (e.g. standard deviation) or associated estimates of uncertainty (e.g. confidence intervals)
☐	☒	For null hypothesis testing, the test statistic (e.g. *F*, *t*, *r*) with confidence intervals, effect sizes, degrees of freedom and *P* value noted<br>*Give P values as exact values whenever suitable.*
☒	☐	For Bayesian analysis, information on the choice of priors and Markov chain Monte Carlo settings
☒	☐	For hierarchical and complex designs, identification of the appropriate level for tests and full reporting of outcomes
☒	☐	Estimates of effect sizes (e.g. Cohen's *d*, Pearson's *r*), indicating how they were calculated

Our web collection on statistics for biologists contains articles on many of the points above.

Software and code

Policy information about availability of computer code

Data collection	Scanimage, with customization for triggering of laser pulses, version scanimage.52.Klioutchnikov.2016-07-05. It should be noted that the added features are now standard for the current release scanimage 'premium' version.<br>For synchronization of microscopy and behavioral data synchronization signals were acquired using Spike 2, version 6.18.
Data analysis	Optical simulations were made using zemax OpticStudio 14.2.<br>Correction for image motion in freely moving experiments was done using custom Matlab software, referenced in the Methods subsection "Alignment of single frames for motion correction".<br>Trace de-noising was carried out using DeepCAD-RT (Git version fe68f28 from Aug 26, 2021, https://github.com/cabooster/DeepCAD-RT)<br>Spike inference was performed using MLspike (https://github.com/MLspike/spikes, %20commit%2032fb84e).<br>All analyses of the data appearing in the manuscript were performed using Matlab, Release 2021a.

For manuscripts utilizing custom algorithms or software that are central to the research but not yet described in published literature, software must be made available to editors and reviewers. We strongly encourage code deposition in a community repository (e.g. GitHub). See the Nature Portfolio guidelines for submitting code & software for further information.

Data

Policy information about availability of data

All manuscripts must include a data availability statement. This statement should provide the following information, where applicable:

- Accession codes, unique identifiers, or web links for publicly available datasets
- A description of any restrictions on data availability
- For clinical datasets or third party data, please ensure that the statement adheres to our policy

Source data available at https://doi.org/10.5061/dryad.76hdr7t11. Zemax files for the optics developed . Zemax files for the optics developed are available on request.

Field-specific reporting

Please select the one below that is the best fit for your research. If you are not sure, read the appropriate sections before making your selection.

☒ Life sciences ☐ Behavioural & social sciences ☐ Ecological, evolutionary & environmental sciences

For a reference copy of the document with all sections, see nature.com/documents/nr-reporting-summary-flat.pdf

Life sciences study design

All studies must disclose on these points even when the disclosure is negative.

Sample size	Imaging data were acquired from 5 different animals as 20 independent datasets. This sample size was deemed sufficient to illustrate the reproducibility of the acquired results, the stability of the miniature imaging system and to provide sufficient data for comparisons of neuronal activity in freely moving mice in lit and darkened conditions, while also considering the ethical use and treatment of the experimental animals.
Data exclusions	Microscopy data with excessive in-frame motion, for example caused by entanglement of the optical fiber and electronic cables, image brightness fluctuation due to shifting of the immersion solution and excessive image motion, were excluded from further analysis. Statistics on the amount of data excluded are presented in the manuscript.
Replication	For microscopy experiments, the results describe 20 imaging datasets acquired in 13 separate imaging sessions from 5 different animals. For comparisons of animal behavior with and without the microscope, data from the above datasets were compared with behavior recorded from 3 naive animals running on the same track without the microscope. For comparison of head orientation, the 6 independent datasets were acquired from 3 animals.
Randomization	For experiments recording neuronal populations in lit and darkened arenas, recordings in lit and dark conditions were conducted in the same imaging period sucessively to ensure recordings in light and dark from the same neurons. For comparison of animal behavior wearing the microscope or only the tracking struts, all animals carried the microscope first then the struts in one session and the struts first then the microscope in another session, with results from all included in the final analysis. The two sessions were separated by a period of one day. Other randomization was not incorporated into the design of the experiments.
Blinding	Experimental blinding was not considered necessary, because distinct experimental groups were not employed and because sensibly achievable.

Reporting for specific materials, systems and methods

We require information from authors about some types of materials, experimental systems and methods used in many studies. Here, indicate whether each material, system or method listed is relevant to your study. If you are not sure if a list item applies to your research, read the appropriate section before selecting a response.

Materials & experimental systems

n/a	Involved in the study	
☒	☐	Antibodies
☒	☐	Eukaryotic cell lines
☒	☐	Palaeontology and archaeology
☐	☒	Animals and other organisms
☒	☐	Human research participants
☒	☐	Clinical data
☒	☐	Dual use research of concern

Methods

n/a	Involved in the study	
☒	☐	ChIP-seq
☒	☐	Flow cytometry
☒	☐	MRI-based neuroimaging

Animals and other organisms

Policy information about studies involving animals; ARRIVE guidelines recommended for reporting animal research

Laboratory animals	The following strains were used:<br>-Neurotensin receptor-1 Cre recombinase line Ntsr1-Cre (B6.FVB(Cg)-Tg(Ntsr1-cre)Gn220Gsat/Mmcd) was obtained from the Mutant Mouse Resource and Research Centers (MMRRC, #030648-UCD).<br>- Sodium channel, nonvoltage-gated 1 alpha-Cre Scnn1a-Cre (Tg(Scnn1a-cre)3Aibs) was obtained from the Jackson Laboratory (#009613). Animals were heterozygous for the transgene with C57BL/6 background.<br>- mice were used that resulted from crossing heterozygous animals of both strains (Ntsr1-Cre/Scnn1a-Cre).<br>- 13 mice were used in these experiments, 11 females and 2 males, aged 8-98 weeks (average 23.5 weeks) at the commencement of the experiment
Wild animals	No wild animals were used in this study.
Field-collected samples	No field-collected samples were used in this study.
Ethics oversight	Landesamt für Natur, Umwelt und Verbraucherschutz Nordrhein-Westfalen (LANUV), Germany

Note that full information on the approval of the study protocol must also be provided in the manuscript.
